# Natural Products for Pancreatitis: Recent Advances, Molecular Mechanisms and Therapeutic Prospects

**DOI:** 10.3390/cells15212002

**Published:** 2026-11-03

**Authors:** Shiyan Su, Jing Yang, Meiyue Zhu, Yunhua Duan, Ruizhe He, Banglan Luo, Lei Tian, Ye Li

**Affiliations:** 1 School of Basic Medicine, Kunming University of Science and Technology, Kunming 650500, China; 2 Faculty of Food Science and Engineering, Kunming University of Science and Technology, Kunming 650500, China

**Keywords:** pancreatitis, natural products, oxidative stress, inflammatory signaling pathways, molecular targets, therapeutic potential

## Abstract

**Highlights:**

**What are the main findings?**

- Natural products offer multi-target, chemically diverse, low-toxicity candidates for pancreatitis prevention and treatment.
- Alkaloids, quinones, terpenes, flavonoids, phenolics, and glycosides are systematically categorized by molecular target and pathway for pancreatitis.

**What is the implication of the main finding?**

- Rational engineering of plant-derived compounds is proposed to accelerate the development of novel pancreatitis therapeutics.
- The multi-target and low-toxicity nature of natural products supports their potential as safer, integrative options for pancreatitis prevention and treatment.

**Abstract:**

Pancreatitis is a prevalent disorder of the digestive system, characterized by the rapid onset of pancreatic exudative edema and acinar cell necrosis. Clinically, the pathogenesis of pancreatitis is multifactorial and involves an intricate interplay among oxidative stress, dysregulated inflammatory signaling pathways, impaired autophagy, and aberrant apoptosis. Currently, no targeted disease-modifying pharmacotherapies are approved for clinical use in pancreatitis. In recent years, natural products have garnered increasing attention due to their inherent advantages, including chemical diversity, multi-target engagement, and favorable safety profiles, rendering them promising candidates for both preventive and therapeutic intervention in pancreatitis. Nevertheless, a comprehensive synthesis of current evidence regarding their mechanisms, bioactive constituents, and translational potential remains lacking. This review provides a rigorously categorized analysis of bioactive natural compounds (including alkaloids, quinones, terpenes, flavonoids, phenolics, and glycosides) and their molecular targets implicated in the prevention and treatment of pancreatitis. Furthermore, it critically evaluates emerging strategies for the rational engineering and optimization of plant-derived compounds, thereby offering a conceptual and mechanistic foundation for the development of novel therapeutics and innovative treatment paradigms for pancreatitis.

## 1. Introduction

**Figure 1:**
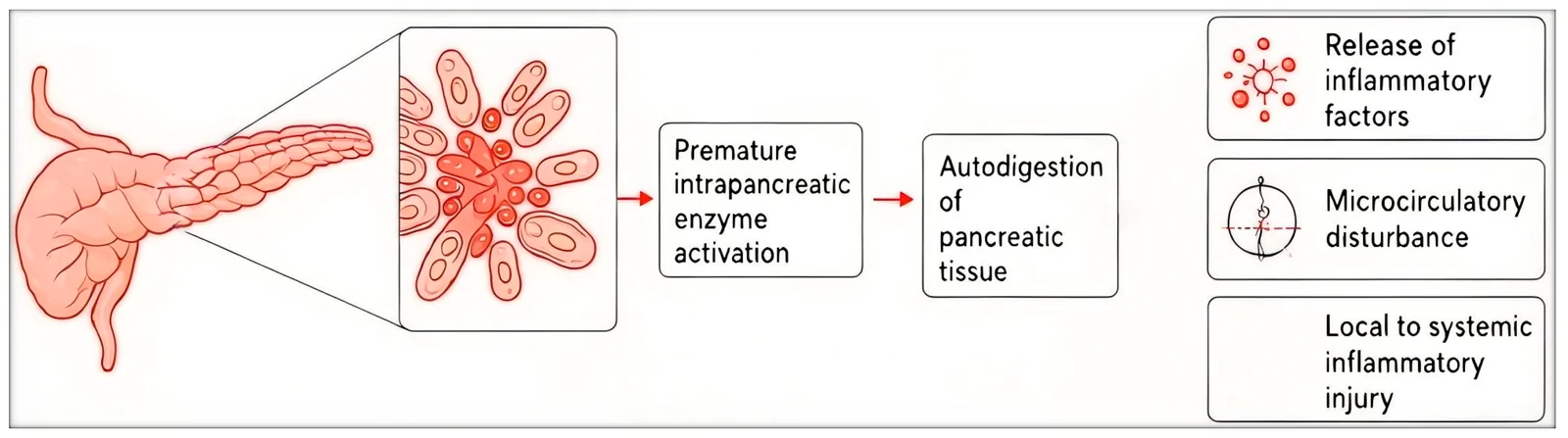
Definition and core pathogenic cascade of pancreatitis. A multifactorial heterogeneous disease.

Pancreatitis refers to a condition in which abnormal activation of pancreatic enzymes leads to autodigestion of the pancreas, release of inflammatory factors, and microcirculatory disturbances; its pathological manifestations include localized or even systemic inflammatory damage to the pancreas, which classifies it as a multifactorial heterogeneous disease [1]. Based on disease course, pancreatitis is categorized into two types: acute pancreatitis (AP) and chronic pancreatitis (CP). AP progresses rapidly, characterized by upper abdominal pain and elevated serum amylase and lipase levels (>3 times the upper limit of normal); it typically has an abrupt onset and rapid progression [2,3]. CP manifests as recurrent episodes of abdominal pain and permanent impairment of both endocrine and exocrine pancreatic functions, with a prolonged disease course that may progress to diabetes mellitus, steatorrhea, or pancreatic cancer [4]. AP may evolve into CP due to chronic inflammation and autodigestion, leading to pancreatic fibrosis and atrophy [5]. Based on etiology, pancreatitis can be classified into biliary pancreatitis (accounting for approximately 50%), alcoholic pancreatitis (approximately 25%), hyperlipidemic pancreatitis, drug-induced pancreatitis, and idiopathic pancreatitis (Figure 1).

**Figure 2:**
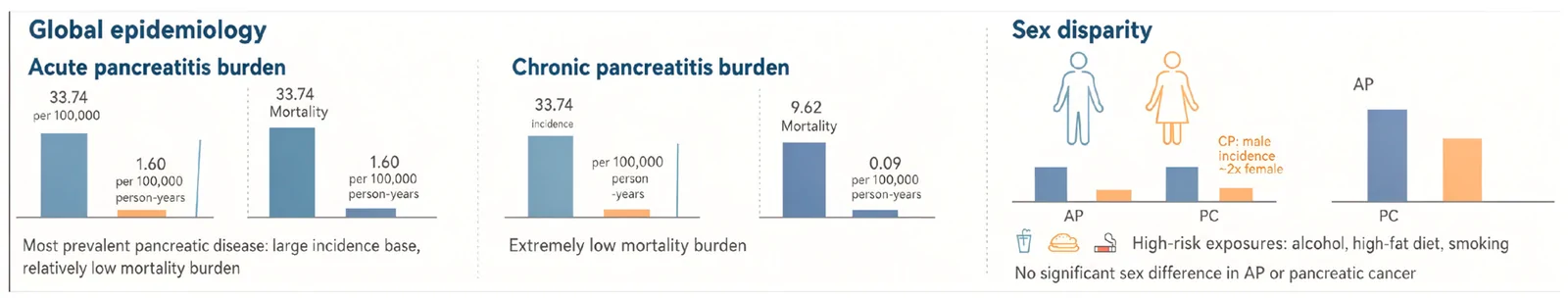
Global epidemiology of pancreatitis. Pancreatitis is a high-incidence, low-mortality, etiologically heterogeneous disease dominated by biliary and alcoholic causes, in which acute injury can evolve into chronic fibrotic disease.

Globally, the incidence rate of AP is approximately 33.74 per 100,000 population per year, showing a consistent upward trend, which makes it the most prevalent pancreatic-related disease. However, the global mortality rate for AP is approximately 1.60 per 100,000 population per year, indicating that AP exhibits epidemiological characteristics of a large baseline incidence but a relatively low mortality burden; gallstones represent the most common etiology of AP [6]. The annual incidence rate of CP is approximately 9.62 per 100,000 population per year, while the global mortality rate is only 0.09 per 100,000 population per year, reflecting an extremely low mortality burden. It is noteworthy that, when analyzed by gender, CP shows significant gender disparities: the incidence rate in males is approximately twice that of females, with alcohol consumption, high-fat diets, and smoking potentially serving as high-risk exposure factors [7]; conversely, no significant gender difference is observed in the incidence rates of both pancreatic cancer (PC) and AP, with overall male and female risk levels being nearly identical (Figure 2).

Treatment regimens differ by pancreatitis type. For AP, supportive care is the mainstay. Initial measures include fasting, gastrointestinal decompression [8], aggressive fluid resuscitation [9], and early enteral nutrition [10]. Nonsteroidal anti-inflammatory drugs (NSAIDs) or opioids are first-line for pain. For biliary pancreatitis with cholangitis, early endoscopic retrograde cholangiopancreatography (ERCP) with stone extraction should be performed [11]. Diagnostic ERCP is not recommended for idiopathic pancreatitis. In high-risk patients, prophylactic rectal indomethacin plus pancreatic duct stenting may be considered [12]. Adjunctive therapies may include somatostatin analogs and urokinase [13]. Antibiotics are used only when infection is confirmed [14]. For pancreatic necrosis or abscess, percutaneous drainage or surgical debridement may be needed [15]. Hyperlipidemic pancreatitis is managed with insulin or plasma exchange. Alcoholic pancreatitis requires abstinence plus psychological intervention. For CP, a multidisciplinary nursing approach addresses pain, endocrine dysfunction, and psychological distress. Endoscopic therapy or pancreaticojejunostomy may be considered for advanced duct obstruction or refractory pain [16]. Emerging techniques favor precision minimally invasive approaches, including targeted drugs against inflammatory cytokines and stem cells with anti-inflammatory and reparative properties [17]. These offer novel therapeutic perspectives for severe and chronic cases.

Natural products are defined as substances with pharmacological or biological properties that are synthesized by natural organisms, including plants, animals, insects, marine organisms, and microorganisms; these compounds refer either to constituent components or metabolites [18,19]. In recent decades, natural products have demonstrated tremendous potential in the treatment of malignant tumors (e.g., liver cancer [20] and breast cancer [21]) and in the regulation of inflammatory responses (e.g., pancreatitis and enteritis) [22]. As primary sources of bioactive compounds, plant-derived natural products have served as precursors for clinical drugs, and played a core role in the development of novel therapeutic agents. Based on structural differences, plant-derived natural products are primarily categorized into alkaloids, phenolics, terpenoids, flavonoids, and glycosides [23]. Existing studies have confirmed that natural products have demonstrated potential in the prevention and treatment of pancreatitis, with their effects primarily manifested in multiple aspects, including anti-inflammatory effects, antioxidant activity, and the regulation of key intracellular signaling pathways.

## 2. Signaling Pathways Implicated in the Pathogenesis of Pancreatitis

### 2.1. Oxidative Stress Pathway

Oxidative stress is a common pathophysiological feature of AP. Known triggers include alcohol consumption, high-fat diets, and biliary stones. These triggers can activate cytochrome P4502E1 (cytochrome P450 family 2 subfamily E member 1, CYP2E1) and NOX (nicotinamide adenine dinucleotide phosphate hydrogen (NADPH) oxidase) in pancreatic acinar cells (PACs). NOX serves as the predominant cellular source of reactive oxygen species (ROS) [24], whereas CYP2E1 catalyzes the oxidative metabolism of both endogenous mediators and exogenous substrates [25]. Oxidative stress is a pathophysiological condition resulting from an imbalance between endogenous antioxidant defenses and ROS, leading to activation of multiple pro-inflammatory signaling pathways and consequent exacerbation of pancreatic injury and systemic damage.

Oxidative stress induces mitochondrial damage, lipid peroxidation, and oxidative DNA lesions. On one hand, mitochondrial DNA (mtDNA) is directly targeted by ROS, leading to oxidative modifications and mutagenic alterations; on the other hand, ROS trigger the release of pro-inflammatory mediators from pancreatic cells, imposing a sustained high metabolic burden on mitochondria and thereby impairing the integrity and function of the mitochondrial respiratory chain [26]. Additionally, mitochondrial dysfunction further amplifies ROS accumulation. An imbalance between pro-oxidant and antioxidant systems promotes intracellular iron overload, which, under the catalytic action of ferrous ions (Fe^2+^) or lipoxygenases, drives lipid peroxidation of membrane-enriched polyunsaturated fatty acids, ultimately overwhelming cellular antioxidant defenses. Moreover, lipid peroxidation serves as the principal driver of ROS generation during iron-dependent apoptosis, establishing a self-amplifying feedback loop between ROS accumulation and lipid peroxidation [27].

In recent years, ROS generated under conditions of oxidative stress have been increasingly recognized not only as harmful byproducts but also as real signaling molecules [28]. These ROS directly promote the activation of macrophages and neutrophils, thereby stimulating the production of pro-inflammatory cytokines-including Interleukin-6 (IL-6), Interleukin-1β (IL-1β), and Tumor Necrosis Factor-alpha (TNF-α) [29]. Concurrently, these cytokines induce endothelial hyperpermeability and impair microcirculatory function, creating a feed-forward loop that further amplifies ROS generation. Moreover, ROS serve as critical upstream initiators of the nuclear factor kappa-B (NF-κB) and mitogen-activated protein kinase (MAPK) signaling pathways [30]. Specifically, ROS facilitate the nuclear translocation of NF-κB, where it undergoes activation and exerts transcriptional regulatory functions. Once activated, NF-κB drives the robust expression and secretion of multiple inflammatory cytokines by macrophages [31]. MAPK represents another critical inflammatory signaling cascade implicated in mediating tissue injury during acute pancreatitis. A bidirectional causal relationship exists between MAPK activation and oxidative stress: MAPK signaling amplifies oxidative stress, while oxidative stress, in turn, further activates MAPK pathways. Within the pancreas, the MAPK family comprises three major subfamilies-p38, c-Jun N-terminal kinase (JNK), and extracellular regulated protein kinases (ERK)1/2. In macrophages, these kinases undergo pathological hyperphosphorylation, triggering robust secretion of pro-inflammatory cytokines and thereby exacerbating disease progression [26,32]. Furthermore, MAPK activation suppresses endogenous antioxidant defense systems that include superoxide dismutase (SOD), glutathione (GSH), and glutathione peroxidase (GPx), an effect that collectively intensifies oxidative stress [33].

Disulfide stress was initially described and characterized within the setting of acute inflammation. This particular form of oxidative stress worsens inflammatory responses by selectively targeting redox-sensitive signaling proteins, such as Keap1, protein phosphatases, apurinic/apyrimidinic endonuclease 1 (APE1)/redox factor-1 (Ref-1), and thioredoxin 1 (Trx1). In contrast to classical oxidative stress, disulfide stress occurs independently of GSH oxidation [34].

**Figure 3:**
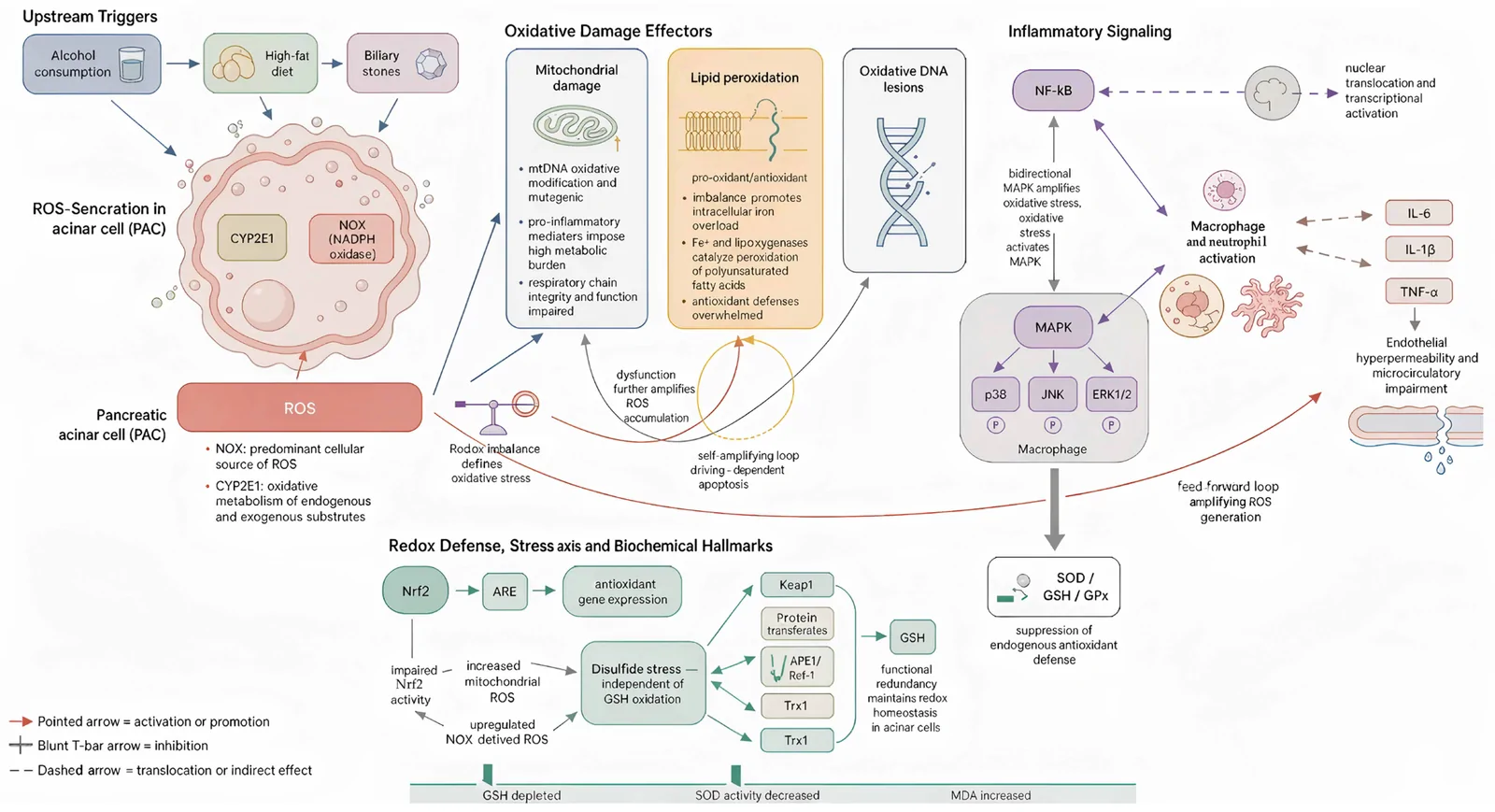
Oxidative stress and redox signaling networks in acute pancreatitis. Alcohol, high-fat diets, and biliary stones activate CYP2E1 and NOX in acinar cells, generating ROS that overwhelm antioxidant defenses and drive mitochondrial damage, lipid peroxidation, and DNA lesions in self-amplifying loops. ROS also activate NF-κB and MAPK in macrophages, inducing IL-6, IL-1β, and TNF-α release, endothelial hyperpermeability, and microcirculatory failure. MAPK suppresses SOD, GSH, and GPx, while disulfide stress targets Keap1, phosphatases, APE1/Ref-1, and Trx1 independently of GSH oxidation. Impaired Nrf2 further elevates ROS.

In pancreatitis, hallmark biochemical alterations include early GSH depletion, diminished SOD activity, and elevated malondialdehyde (MDA) levels [35]. Notably, Trx1 induction constitutes a key antioxidant defense mechanism: it functions analogously to GSH in protecting pancreatic acinar cells, and the two systems exhibit functional redundancy-capable of compensating for each other to maintain redox homeostasis [36]; nuclear factor erythroid 2-related factor 2 (Nrf2) is a master transcriptional regulator of antioxidant responses, and its impaired activity promotes ROS accumulation via two major pathways: enhanced mitochondrial ROS production and upregulated NOX-derived ROS generation [22] (Figure 3).

Natural products such as flavonoids and polyphenolic compounds can ameliorate oxidative damage associated with pancreatitis through multiple mechanisms: direct scavenging of ROS, activation of the Nrf2/ARE signaling pathway, and suppression of pro-inflammatory signaling cascades [37]. For instance, the seed coat of purple rice demonstrates potent antioxidant capacity: its principal bioactive constituents, such as anthocyanins, flavonoids, and phenolic acids, effectively neutralize oxygen-derived free radicals in vivo [38]; similarly, Qingyi Decoction and its pharmacologically active components alleviate AP by modulating acinar cell function and macrophage polarization via inhibition of the NF-κB/NOD-like receptor thermal protein domain-associated protein 3 (NLRP3)/Caspase-1 inflammasome axis [39]; Dahuang Mudan Decoction (including ogonoside, catechins, rhein) has been shown to attenuate pancreatic oxidative injury by concurrently targeting oxidative stress and the MAPK signaling pathway-specifically, by suppressing MAPK phosphorylation [32]. Furthermore, shikonin exerts protective effects against caerulein-induced experimental pancreatitis by inhibiting NF-κB activation, thereby reducing the secretion of downstream pro-inflammatory cytokines [40]. Collectively, these findings underscore targeting oxidative stress-related pathways as a promising strategy for the prevention and treatment of pancreatitis.

### 2.2. Inflammatory Pathways

In AP, damage-associated molecular patterns (DAMPs) (e.g., HMGB1 and histones) released by necrotic PACs and pathogen-associated molecular patterns (PAMPs) (e.g., Lipopolysaccharides (LPS)) derived from displaced gut microbiota jointly activate Toll-like receptor 4 (TLR4). TLR4 activates NF-κB via the myeloid differentiation primary response protein 88 (MyD88) pathway, and activates signal transducer and activator of transcription 3 (STAT3) via the TIR domain-containing adaptor inducing interferon-β (TRIF) pathway. Furthermore, STAT3 can reciprocally augment NF-κB activity, forming a positive feedback loop that amplifies inflammatory responses [41]. This axis drives the massive release of TNF-α, IL-1β, IL-6, monocyte chemoattractant protein-1 (MCP-1/CCL2), and IL-8, which in turn induce neutrophil and macrophage infiltration, exacerbate pancreatic necrosis, and trigger systemic inflammatory response syndrome (SIRS). This constitutes the core mechanism underlying the progression of pancreatitis from localized injury to multi-organ failure. After TLR4 initiates inflammation, infiltrating neutrophils and macrophages in pancreatic tissue release matrix metalloproteinases (MMPs) that degrade the extracellular matrix (ECM) and disrupt the tissue structural scaffold, while these cells concurrently generate ROS that exert direct cytotoxic damage to PACs [41]. NF-κB upregulates NLRP3 expression and affects inflammasome. When DAMPs function as a secondary signal and translocate to the Golgi apparatus, the inflammasome assembles and becomes activated, which cleaves pro-IL-1β into active IL-1β and induces pyroptosis. DAMPs released upon pyroptotic cell lysis subsequently activate TLR4, forming a positive feedback loop. The combined effects of MMPs, ROS, and the NLRP3/pyroptosis pathway escalate local inflammation to tissue necrosis, microcirculatory dysfunction, and SIRS, propelling pancreatitis toward multiple organ failure. During this process, neutrophil extracellular traps (NETs) directly damage the vascular endothelium via their histones and elastase components, and concurrently activate the coagulation system to promote microthrombus formation, which further exacerbates pancreatic ischemia and necrosis [42].

Terpenoids and alkaloids, as natural products, can target multiple stages of the aforementioned inflammatory cascade simultaneously. They can inhibit the nuclear translocation of NF-κB, thereby blocking the transcription of pro-inflammatory genes [43]; they can also inhibit phosphorylation in the MAPK pathway to attenuate the amplification of the inflammatory cascade. Additionally, these compounds downregulate the expression of the NLRP3 inflammasome, reducing the maturation and release of pro-inflammatory cytokines such as IL-1β. Certain terpenoid alkaloids can also activate the aryl hydrocarbon receptor (AHR) pathway, producing a synergistic anti-inflammatory effect [44].

### 2.3. Autophagy

Autophagy serves as a core degradation mechanism through which PACs maintain homeostasis; the disruption of the autophagy–lysosome pathway integrity represents a hallmark molecular pathological alteration in pancreatitis. Autophagy deficiency significantly reduces the degree of trypsinogen activation induced by caerulein, suggesting that autophagy mediates intracellular activation of trypsinogen by delivering it to the lysosomal lumen, and this process constitutes an essential subcellular structural foundation for proenzyme activation during the early stages of pancreatitis [45]. Similarly, the integrity of the autophagy flux is critically important. Defects in the lysosome-associated membrane protein 2 (LAMP-2) can lead to impaired fusion between autophagosomes and lysosomes, as well as reduced degradation capacity, resulting in vacuolation of PACs, inflammatory infiltration, and fibrosis [46].

Alcohol and high-fat diets are significant triggers of pancreatitis, both contributing to disease pathogenesis by inhibiting autophagy flux. Alcohol intake induces lipid droplet accumulation within the pancreas, simultaneously activates the AMPK signaling pathway, and impairs autophagy flux during AP, thereby upregulating anti-apoptotic proteins, suppressing apoptosis, and enhancing necrosis [47,48]. In contrast, a high-fat diet indirectly impairs autophagy efficiency through lipid metabolism disorders and mitochondrial dysfunction, leading to the accumulation of damaged mitochondria and activated trypsinogen within the cell, thereby further inducing acinar cell necrosis [49]. The accumulation level of autophagy-related proteins such as Beclin-1, LC3-II, and p62 directly reflects the degree of impairment in the autophagy-mediated degradation function. Combined analysis of these three parameters provides a comprehensive approach for evaluating the functional status of autophagy at different stages of pancreatitis.

Recent studies have revealed that natural products such as glycosides and phenols can exert pancreas-protective effects by restoring the autophagy flux. This mechanism involves the regulation of the AMPK/mTOR pathway: specifically, the natural products upregulate the LC3-II/I ratio and reduce p62 protein levels to restore the integrity of the autophagy flux, thereby accelerating the clearance of damaged mitochondria and activated proenzymes, and alleviating acinar cell necrosis and inflammatory responses [46]. It should be noted that the role of autophagy in pancreatitis is context-dependent and cannot be reduced to a simple “more is better” paradigm. In experimental pancreatitis, autophagy is activated but its flux is impaired at the lysosomal degradation step, and this impaired flux—rather than a mere deficiency of autophagic activity—contributes to intra-acinar trypsinogen activation and vacuole accumulation [47]. Notably, genetic ablation of autophagy reduces trypsinogen activation in early pancreatitis [45], indicating that autophagic delivery to lysosomes can paradoxically promote proenzyme activation when lysosomal degradation capacity is compromised. Therefore, the therapeutic significance of natural products that modulate autophagy should be interpreted in terms of their effects on flux integrity and stage-specific action, not merely on LC3-II levels or autophagic “activity.” A compound that enhances autophagosome formation without restoring lysosomal degradation may aggravate rather than alleviate acinar injury.

### 2.4. Apoptosis

During the progression of pancreatitis, apoptosis of pancreatic acinar cells is primarily mediated through three core signaling pathways: the mitochondrial intrinsic pathway, the death receptor extrinsic pathway, and the endoplasmic reticulum (ER) stress pathway [50]. Cytoplasmic calcium overload, accumulation of ROS, and premature activation of intracellular pancreatic enzymes all serve as upstream triggers for the activation of these pathways [51,52].

In the inflammatory microenvironment, TNF-α and FasL bind to the TNFR1 and Fas receptors on acinar cell membranes, assembling the death-inducing signaling complex (DISC) that activates caspase-8; this subsequently cleaves Bid to generate tBid, which translocates to the mitochondria [53]. Simultaneously, persistent calcium overload opens the mitochondrial permeability transition pore (mPTP), leading to collapse of the mitochondrial membrane potential and release of cytochrome C into the cytosol, where it binds to Apaf-1 to activate caspase-9. These two signaling pathways collectively activate the downstream execution molecule caspase-3/7, thereby inducing orderly apoptosis [54]. Meanwhile, the endoplasmic reticulum stress pathway upregulates the pro-apoptotic protein Bax via the C/EBP homologous protein (CHOP) and JNK signaling cascades, thereby amplifying the mitochondrial damage signal [55].

**Figure 4:**
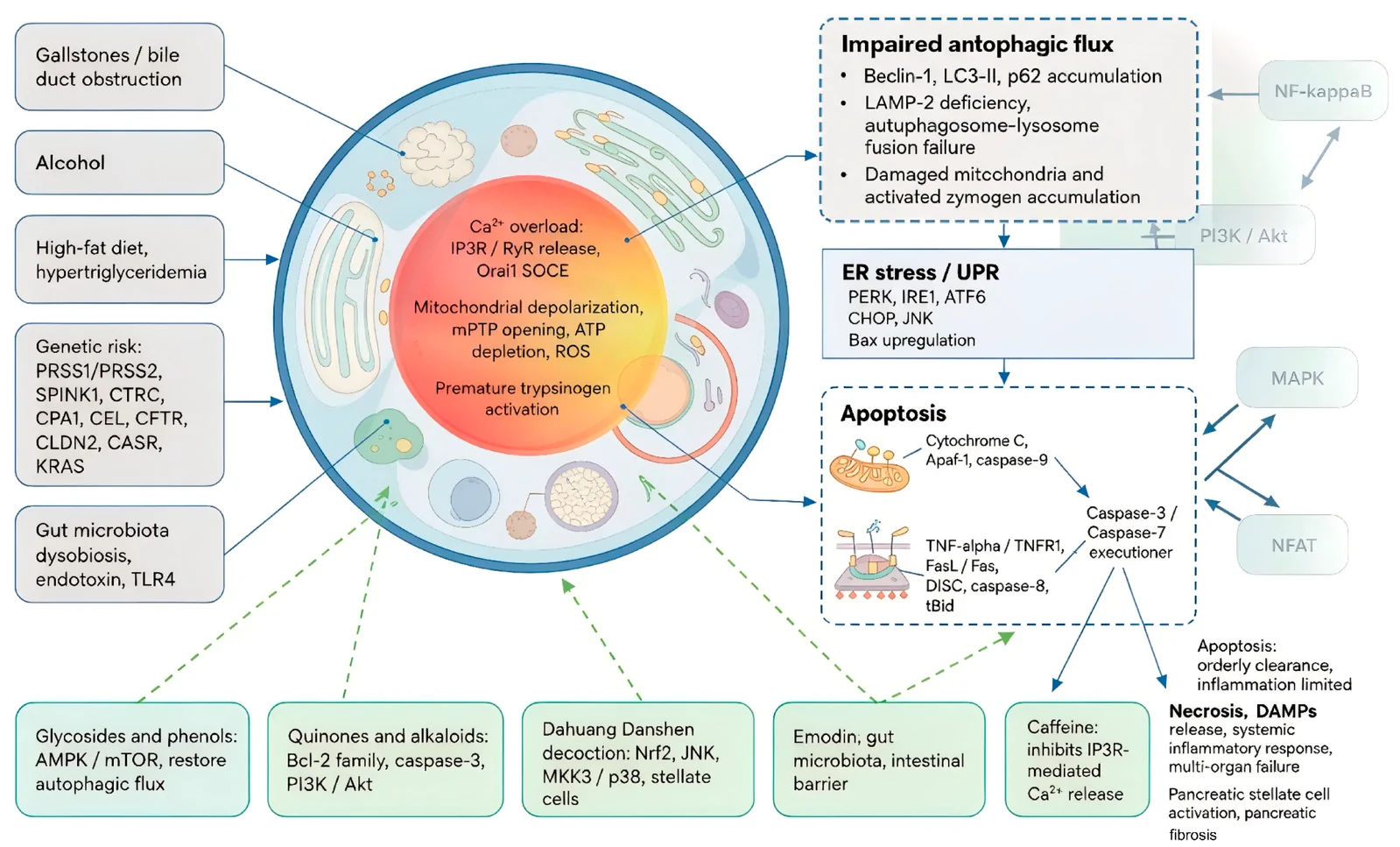
Cell death networks in acute pancreatitis. Etiological insults disrupt autophagic flux and provoke ER Ca^2+^ release via IP3R/RyR and Orai1, causing mitochondrial depolarization, ATP loss, ROS accumulation and premature trypsinogen activation in pancreatic acinar cells. These events engage UPR and intrinsic and extrinsic apoptotic pathways; severe injury shifts apoptosis to necrosis with DAMP-driven systemic inflammation. Natural products targeting AMPK/mTOR, Bcl-2/caspase-3, Nrf2 and gut microbiota restore homeostasis. Arrows denote activation; flat bars denote inhibition.

In mild pancreatitis, apoptosis of pancreatic acinar cells occurs predominantly, clearance of damaged cells can help limit the spread of inflammation. In severe pancreatitis, mitochondrial damage is severe, adenosine triphosphate (ATP) depletion is extremely pronounced, the apoptotic signaling pathway becomes impaired, and cell death shifts toward necrosis; the release of numerous DAMPs triggers a systemic inflammatory response [22]. Alternative pathways such as NF-κB, phosphatidylinositol 3-kinase (PI3K)/Akt, and MAPK can bidirectionally regulate the balance of these apoptotic pathways, thereby serving as key molecular targets for the grading of pancreatitis severity and for targeted therapeutic interventions [56] (Figure 4).

Quinones and alkaloids, as highly bioactive natural small-molecule compounds, modulate the apoptotic program in pancreatic acinar cells by targeting multiple signaling pathways [57]. Mechanistically, they regulate the expression of Bcl-2 family proteins, moderately activate caspase-3, and suppress the PI3K/Akt signaling cascade, which facilitates programmed apoptosis of injured acinar cells and mitigates necrotic cell death [58]. In addition, these compounds dampen excessive inflammatory activation and block inflammatory cascade amplification, thereby alleviating pancreatic edema, inflammatory infiltration, and local inflammatory spread, and ultimately ameliorating pancreatitis-associated pathological injuries [22,59].

### 2.5. Other Factors

The endoplasmic reticulum (ER) serves as a primary intracellular store of calcium ions (Ca^2+^) store in PACs. Under pathological conditions, inositol 1,4,5-trisphosphate receptors (IP3Rs) and ryanodine receptors (RyRs) on the ER become aberrantly activated, triggering massive Ca^2+^ release into the cytoplasm. Concurrently, depletion of ER Ca^2+^ reserves activates the calcium release-activated calcium channel protein 1 (Orai1), further elevating cytoplasmic Ca^2+^ levels [60]. This sustained Ca^2+^ overload ultimately activates the NF-κB and nuclear factor of activated T cell (NFAT) pathways, leading to ATP depletion and subsequent pancreatic tissue damage [61,62]. Notably, caffeine has been reported to inhibit Ca^2+^ release induced by certain subtypes of IP3, thereby preventing mitochondrial depolarization and PACs necrosis [63].

Under ER stress, the unfolded protein response (UPR) continuously activates apoptotic pathways, such as the JNK pathway, ultimately driving cell death [64,65]. Studies have shown that the Dahuang Danshen Decoction upregulates the expression of Nrf2-downstream genes, suppresses JNK and MKK3/p38 signaling, and inhibits the activation of pancreatic stellate cells (PSCs), thereby exerting a protective effect against pancreatic fibrosis (PF) [66].

The onset and progression of pancreatitis are also tightly linked to the regulation of genetic pathways. In the trypsin pathway, mutations in serine protease 1/2 (PRSS1/2) cause excess enzyme production, while mutations in serine peptidase inhibitor, Kazal type 1 (SPINK1) or chymotrypsin C (CTRC) cause abnormal activation, triggering autodigestion [67]. In the protein misfolding pathway, mutations in PRSS1, carboxypeptidase A1 (CPA1) or carboxyl ester lipase (CEL) cause digestive enzymes to misfold, triggering ER stress [68]. In the pancreatic ductal pathway, mutations in cystic fibrosis transmembrane conductance regulator (CFTR), claudin-2 (CLDN2), or calcium-sensing receptor (CASR) impair ductal ion transport and barrier function [69]. In the fibrosis pathway, mutations in the KRAS proto-oncogene (KRAS) activate PSCs, driving fibrosis and malignant transformation [70]. Beyond these mechanisms, gut microbiota dysbiosis in severe acute pancreatitis (SAP) disrupts tight junction proteins, increases intestinal permeability, and promotes bacterial endotoxin translocation; this exacerbates the systemic inflammatory response via the TLR4 pathway [71]. Emodin class natural products have been shown to modulate the gut microbiota composition and repair the intestinal barrier, thereby improving prognosis [72,73].

## 3. Application of Natural Products in the Prevention and Treatment of Pancreatitis

### 3.1. Alkaloids

#### 3.1.1. Berberine (C_20_H_18_NO_4_^+^)

Berberine, commonly known as “huangliansu” in traditional Chinese medicine, is a pentacyclic isoquinoline alkaloid and quaternary ammonium compound found in abundance in medicinal plants such as *Coptis chinensis* Franch., *Berberis vulgaris*, and *Phellodendron amurense* (Table 1). Berberine exhibits potent anti-inflammatory, antioxidant, antidiabetic, and anticancer activities across multiple tissues and organ systems. However, oral administration of Berberine is associated with poor intestinal absorption and extremely low systemic bioavailability; consequently, its pharmacological effects are increasingly attributed to its biotransformation products rather than the parent compound [74]. Accumulating evidence indicates that Berberine mitigates cellular damage, oxidative stress, and inflammation in pancreatic β-cells—effects that are potentially mediated by downregulation of the NF-κB and JNK signaling pathways [75,76].

#### 3.1.2. Matrine (C_15_H_24_N_2_O) and Oxymatrine (C_15_H_24_N_2_O_2_)

These are quinoline alkaloids isolated from the dried roots, stems, and fruits of *Sophora flavescens*, a leguminous plant, via extraction with organic solvents—most commonly ethanol (Table 1). Oxymatrine has emerged as a prominent focus of current pharmacological research: accumulating evidence indicates that it confers organ- and tissue-protective effects through modulation of inflammatory responses, oxidative stress, apoptosis, and fibrotic processes [77]. Nevertheless, the clinical translation of matrine remains hindered by several pharmacokinetic and safety challenges, including low oral bioavailability, limited chemical stability, and dose-dependent hepatotoxicity and neurotoxicity [78]. Structurally related to matrine and often used interchangeably with it in the literature, oxymatrine demonstrated therapeutic efficacy in an L-arginine (Arg)-induced acute pancreatitis model in preclinical studies, ameliorating both pancreatic injury and associated intestinal damage by suppressing pro-inflammatory cytokine production and inhibiting the MAPK/NF-κB signaling cascade [79]. Moreover, matrine has shown promise in mitigating severe acute pancreatitis-associated acute lung injury (SAP-ALI), potentially via activation of the uncoupling protein 2 (UCP2)/sirtuin 3 (SIRT3)/peroxisome proliferator-activated receptor gamma coactivator 1-alpha (PGC1α) mitochondrial regulatory axis [80].

#### 3.1.3. Ellipticine (C_17_H_14_N_2_)

Ellipticine is a cytotoxic alkaloid naturally occurring in plants of the Apocynaceae family. It is found in high concentrations in the leaves of *Ochrosia elliptica* Labill., as well as in the bark and leaves of *Ochrosia balansae* and *Ochrosia moorei* (Table 1). Ellipticine exhibits well-documented antitumor activity, primarily through induction of cancer cell apoptosis via modulation of cell cycle progression and key apoptotic signaling pathways [81]. Moreover, accumulating evidence supports its anti-inflammatory properties. Recent studies suggest that ellipticine exerts a synergistic inhibitory effect on IL-17A and TNF-α, indicating its potential as a therapeutic agent for acute severe pancreatitis [82].

#### 3.1.4. Evodiamine (C_19_H_17_N_3_O)

Evodiamine is an indolequinazoline alkaloid isolated from the dried, unripe fruits of *Evodia rutaecarpa* (Juss.) Benth., a medicinal plant belonging to the Rutaceae family (Table 1). Despite its broad pharmacological potential, evodiamine suffers from suboptimal physicochemical properties, including low aqueous solubility and limited oral bioavailability, as well as only moderate intrinsic biological activity. Traditionally, it has been documented to exert anti-inflammatory, antiplatelet, vasodilatory, analgesic, antitumor, and antidiabetic effects [83]. Notably, evodiamine demonstrates a pronounced therapeutic effect in caerulein-induced AP, and its primary mechanism involves modulation of the calcitonin gene-related peptide (CGRP) pathway: evodiamine upregulates CGRP expression, thereby selectively suppressing excessive intracellular activation of the MAPK and NF-κB signaling cascades [84,85]. Moreover, emerging evidence positions evodiamine as a promising scaffold for the development of novel antimicrobial agents, particularly to address the challenge of antibiotic resistance [86].

#### 3.1.5. Capsaicin (C_18_H_27_NO_3_)

The primary bioactive compound in chili peppers, capsaicin, is a vanillylamide alkaloid and a well-characterized agonist of the transient receptor potential vanilloid 1 (TRPV1) channel (Table 1). Extensive preclinical evidence supports its diverse pharmacological properties, including anticancer, anti-inflammatory, anti-obesity, antioxidant, analgesic, and neuroprotective effects against Alzheimer’s disease [87]. With respect to its anti-inflammatory activity, mechanistic studies indicate that capsaicin attenuates inflammatory responses by ameliorating intracellular lipid accumulation. Moreover, it exerts synergistic anti-inflammatory effects through two regulatory pathways: TRPV1-mediated neuroimmune modulation and peroxisome proliferator-activated receptor gamma (PPARγ)-dependent transcriptional regulation, both converging to suppress the production and release of pro-inflammatory cytokines [88].

**Table 1:** Selected Alkaloids in Acute Pancreatitis: Major Sources and Mechanisms of Action.

Natural Products	Major Sources	Mechanisms	Reference
Berberine	*Coptis chinensis, Berberis vulgaris, Phellodendron amurense*	Inhibits NF-κB nuclear translocation Inhibits JNK signaling pathways	[74,75,76]
Matrine and oxymatrine (OMT)	*dried roots, stems, and fruits of Sophora flavescens*	Inhibits the MAPK/NF-κB signaling cascade Suppresses pro-inflammatory cytokine production Activates the UCP2/SIRT3/PGC1α Mitochondrial Regulatory Axis to alleviate SAP-ALI	[77,79,80]
Ellipticine	*Ochrosia elliptica Labill.,* *Ochrosia balansae,* *Ochrosia moorei*	Combines inhibition of IL-17A and TNF-α Induces cancer cell apoptosis via cell cycle/apoptotic pathway	[81,82]
Evodiamine	*Evodia rutaecarpa (Juss.) Benth.*	Upregulates CGRP, selectively inhibiting MAPK/NF-κB hyperactivation.	[84,85]
Capsaicin	*chili peppers*	Inhibits intracellular lipid accumulation Suppression of pro-inflammatory cytokine production/release via combined TRPV1 neuroimmune and PPARγ transcriptional regulation	[87,88]

### 3.2. Quinones

#### 3.2.1. Emodin (C_15_H_10_O_5_)

Emodin is a hydroxyanthraquinone compound naturally occurring in the rhizomes of several plant species, including *Rheum palmatum* L. (a member of the Polygonaceae family), *Aloe vera*, and *Angelica sinensis* (Table 2). In recent years, its broad spectrum of pharmacological activities, such as anticancer, anti-inflammatory, antioxidant, antibacterial, antiviral, and hypoglycemic effects, have attracted increasing scientific attention [89,90,91]. In the context of pancreatic inflammation, emodin exerts its therapeutic effects primarily through anti-inflammatory and antioxidant mechanisms: specifically, it suppresses NLRP3 inflammasome activation by downregulating the expression of key associated proteins such as voltage-dependent anion channel 1 (VDAC1), NLRP3, caspase-1, and IL-18, thereby attenuating downstream inflammatory signaling pathways [92]. Furthermore, emodin inhibits the nuclear translocation of NF-κB and reduces the production of pro-inflammatory cytokines, such as IL-6 and TNF-α [93]. In addition, emodin modulates oxidative stress via four complementary mechanisms: (i) direct scavenging of reactive oxygen species; (ii) upregulation of endogenous antioxidant defense systems, including SOD and GPx; (iii) suppression of inflammation-driven oxidative damage; and (iv) chelation of redox-active metal ions essential for oxidative stress propagation [94]. However, the clinical application of emodin has long been hindered by its low intrinsic bioactivity and dose-limiting toxicities associated with oral administration, including nephrotoxicity, hepatotoxicity, and genotoxicity [95]. In recent years, advances in nanodelivery technologies have enabled the development of a novel rhubarb-derived emodin nanoformulation, which facilitates selective accumulation of emodin in pancreatic and gastrointestinal tissues. This targeted delivery strategy mitigates systemic toxicity while significantly enhancing oral bioavailability [96]. Furthermore, an ultrasound-assisted crystallization technique has been established to fabricate innovative emodin-based co-crystals with complementary natural products, thereby harnessing synergistic pharmacological effects and addressing key barriers to clinical translation [97].

#### 3.2.2. Shikonin (C_16_H_16_O_5_)

A fat-soluble, purplish-red naphthoquinone pigment naturally occurring in the roots of *Lithospermum erythrorhizon* and *Lithospermum sinense* (both members of the *Boraginaceae* family), with shikonin and its derivatives constituting its principal bioactive constituents (Table 2). Recent studies demonstrate that n-hexane-extracted shikonin exhibits inhibitory activity against α-amylase and pancreatic lipase, indicating promising antioxidant, antidiabetic, and antihyperlipidemic properties [94]. In the context of pancreatic cancer therapy, shikonin has been shown to potentiate the efficacy of conventional chemotherapy by suppressing PAK1 activation and its downstream signaling cascade, thereby inhibiting pancreatic cancer cell proliferation and promoting apoptosis [98]. In research on pancreatitis treatment, studies have demonstrated that shikonin attenuates acute pancreatitis in a mouse model induced by caerulein, primarily through inhibition of the NF-κB signaling pathway [99]. Moreover, shikonin may ameliorate acute inflammation by modulating gut microbiota composition and suppressing the expression of pro-inflammatory cytokines, including TNF-α, IL-6, and IL-1β [100]. Structural optimization, which is achieved via chemical modification of the naphthazarin core or side chains, yields novel shikonin derivatives with enhanced biological activity and reduced toxicity [101].

#### 3.2.3. Tanshinone IIA (C_19_H_18_O_3_)

Tanshinone IIA (TanIIA) is a diterpenoid quinone isolated from the dried roots and rhizomes of *Salvia miltiorrhiza* Bunge (Lamiaceae). TanIIA demonstrates diverse pharmacological activities, including anti-inflammatory, antioxidant, cardioprotective, and antitumor effects (Table 2). However, its therapeutic application is hindered by low natural abundance, poor aqueous solubility, and limited oral bioavailability. Consequently, structural modification strategies aimed at enhancing its pharmacokinetic and pharmacodynamic properties have emerged as a major focus in current pharmaceutical research [102]. Studies have reported that TanIIA modulates the IL-6/JAK2/STAT3 signaling pathway, thereby downregulating pro-inflammatory cytokines while upregulating the anti-inflammatory cytokine IL-10. Moreover, by promoting macrophage polarization toward an anti-inflammatory phenotype, TanIIA ameliorates the inflammatory microenvironment within pancreatic islets [103]. In a murine model of acute pancreatitis, TanIIA has been shown to attenuate oxidative stress, this effect is likely mediated through activation of the Nrf2/HO-1 pathway, leading to reduced ROS production and mitigation of mitochondrial damage [104]. In the context of pancreatic cancer, emerging evidence indicates that tanshinone compounds, including TanIIA, can paradoxically elevate intracellular ROS levels by activating the AKT/forkhead b ox O3 (FOXO3)/Superoxide dismutase (SOD2) signaling axis, thereby suppressing tumor cell proliferation and inducing apoptosis [103]. In addition, researchers structurally modified the peripheral methyl groups of TanIIA and cryptotanshinone by converting them into amide functionalities, thereby identifying a novel NLRP3 inflammasome inhibitor featuring TanIIA as a core structural component, offering a promising new therapeutic strategy for inflammatory diseases [105].

#### 3.2.4. Juglone (C_10_H_6_O_3_)

Juglone is a naphthoquinone compound naturally present in the fresh, ripe husks, roots, leaves, and bark of walnut trees [106]. Juglone exhibits a broad spectrum of pharmacological activities, including antibacterial [107], anticancer [108,109,110], anti-inflammatory, and antioxidant effects [111] (Table 2). Although studies specifically investigating juglone’s role in pancreatitis remain scarce, accumulating evidence supports its therapeutic potential in various inflammatory conditions, such as respiratory inflammation [112], inflammatory bowel disease [113], and prostatitis [111]. Mechanistically, juglone’s anti-inflammatory properties are thought to involve suppression of the NLRP3 inflammasome, downregulation of the NF-κB signaling pathway, and modulation of oxidative stress. In the treatment of pancreatic cancer, juglone exhibits three distinct, mechanistically supported anticancer activities: (i) as a potent Pin1 inhibitor, it overcomes therapeutic resistance in pancreatic ductal adenocarcinoma (PDAC) [114]; (ii) as a monotherapy, it induces ferroptosis-mediated apoptosis in pancreatic cancer cells [115]; and (iii) by suppressing the Wnt/β-catenin signaling pathway, it inhibits tumor angiogenesis and metastasis [116]. However, native juglone is limited by poor aqueous solubility (0.04 mg/mL) and by sparse in vivo pharmacokinetic and efficacy data [117]. To address these limitations, recent advances have yielded micelle-based nanocarrier formulations of juglone—designed to enhance solubility, improve tumor accumulation, and enable microenvironment-modulated, targeted delivery to pancreatic tumors [118].

**Table 2:** Selected Quinones in Acute Pancreatitis: Major Sources and Mechanisms of Action.

Natural Products	Major Sources	Mechanisms	Reference
Emodin	*Rheum palmatum L.* *Aloe vera* *Angelica sinensis*	Suppression of NLRP3 inflammasome activation via downregulation of VDAC1, NLRP3, caspase-1, and IL-18 Inhibits NF-κB nuclear translocation Inhibits expression of IL-6 and TNF-α (pro-inflammatory cytokines). Regulating Oxidative Stress: removing ROS; upregulating endogenous antioxidant defense systems; suppressing inflammation-driven oxidative damage; chelating redox-active metal ions	[92,93,94]
Shikonin	*Lithospermum erythrorhizon* *Lithospermum sinense*	Inhibits NF-κB signaling pathway Modulates gut microbiota composition Suppresses the expression of pro-inflammatory cytokines-including TNF-α, IL-6, and IL-1β Inhibits the activation of PAK1 and its downstream signaling cascades, thereby suppressing pancreatic cancer cell proliferation	[98,99,100]
Tanshinone IIA (TanIIA)	*Salvia miltiorrhiza Bunge*	Modulates the IL-6/JAK2/STAT3 signaling pathway, downregulates the expression of pro-inflammatory cytokines Activates the polarization of macrophages toward an anti-inflammatory phenotype Activates the Nrf2/HO-1 pathway curtails ROS production and attenuates mitochondrial damage Activates the AKT/FOXO3/SOD2 signaling axis, inhibits tumor cell proliferation, and induces apoptosis	[103,105]
Juglone	*walnut trees*	Inhibits NLRP3 inflammasome Downregulation of the NF-κB signaling pathway Modulation of oxidative stress Anticancer activity: Inhibits the Pin1 protein to overcome treatment resistance in PDAC; induces ferroptosis in pancreatic cancer cells; inhibits the Wnt/β-catenin signaling pathway, thereby suppressing tumor angiogenesis and metastasis	[106]

### 3.3. Terpenoids

#### 3.3.1. Triptolide (C_20_H_24_O_6_)

Triptolide is a diterpenoid lactone compound isolated from the roots of *Tripterygium wilfordii* Hook. f. (*Celastraceae*) (Table 3). As a traditional Chinese medicine, *Tripterygium wilfordii* has long been used to resolve dampness and dredge collaterals to relieve pain, with primary applications in treating rheumatoid arthritis, nephritis, and autoimmune diseases [114,119]. Recent studies have shown that triptolide exhibits potent anti-inflammatory and immunosuppressive activities [120] and has demonstrated pharmacological potential in multiple fields, including anti-tumor and anti-fibrotic effects [121]. In the context of pancreatitis, triptolide primarily alleviates pancreatic inflammatory cell infiltration and fibrosis by inhibiting STAT3 phosphorylation and reducing IL-6 production [122]. However, its clinical application is severely limited by pronounced hepatotoxicity and reproductive toxicity, so it is mainly used as a lead compound for structural modification and mechanistic research [123].

#### 3.3.2. Paeoniflorin (C_23_H_28_O_11_)

Paeoniflorin is a monoterpene glycoside compound extracted from the roots of *Paeonia lactiflora* Pall. (Paeoniaceae), and serves as the primary bioactive component in both white peony root and red peony root (Table 3). It is mainly produced in regions such as Zhejiang, Anhui, and Sichuan provinces of China, with a long history of medicinal use dating back to Shennong’s Classic of Materia Medica (Shennong Bencao Jing). In traditional Chinese medicine, peony root is commonly used to nourish the blood, regulate menstruation, soothe the liver, and relieve pain, primarily for treating blood deficiency, menstrual irregularities, and hypochondriac pain [124]. Recent studies have shown that paeoniflorin exhibits significant anti-inflammatory, anti-tumor, neuroprotective, cardioprotective, and hepatoprotective activities [125]. In AP, paeoniflorin exerts its therapeutic effect by specifically inhibiting the calcium-activated chloride channel TMEM16A, thereby blocking chloride efflux driven by calcium overload. This mechanism reduces the phosphorylation of NF-κB and decreases the release of pro-inflammatory cytokines (TNF-α and IL-1β), ultimately alleviating pancreatic edema and necrosis [126]. Paeoniflorin also ameliorates pancreatitis-associated kidney and lung injury: in the kidney, it downregulates the HMGB1/TLR4 signaling pathway and inhibits renal tubular epithelial apoptosis [127]; in the lung, it suppresses NF-κB and MAPK phosphorylation in lung tissue and protects the microvascular endothelial barrier [128]. These effects collectively contribute to its protective role against extrapancreatic organ damage in AP.

#### 3.3.3. Glycyrrhizic Acid (C_42_H_62_O_16_)

Glycyrrhizic acid is a primary triterpenoid saponin component found in the roots and rhizomes of *Glycyrrhiza uralensis* Fisch. ex DC. (Fabaceae) (Table 3). It exhibits multifaceted pharmacological effects, including anti-inflammatory, antiviral, and antibacterial activities, and also has a natural sweet taste [129]; consequently, it is now widely used in the medical and food industries. In pancreatitis, its mechanism of action is relatively broad: glycyrrhizic acid directly binds to HMGB1, blocks its interaction with TLR4, and inhibits inflammatory cascades [130]; additionally, it alleviates primary pancreatic cell damage by inhibiting the MAPK/STAT3/AKT signaling pathway [131], specifically by downregulating the excessive phosphorylation of kinases such as p38, JNK, STAT3, and AKT, thereby reducing PACs apoptosis, necrosis, and vacuolization; furthermore, it inhibits ferroptosis via the Keap1/Nrf2/heme oxygenase-1 (HO-1) pathway, which promotes the nuclear translocation of Nrf2, thereby upregulating HO-1, scavenging lipid ROS, and blocking lipid peroxidation, thus alleviating myocardial ferroptosis and suggesting potential therapeutic value in acute pancreatitis-induced cardiac injury [132]. Meanwhile, glycyrrhizic acid can also activate the Nrf2-ARE pathway to promote the expression of detoxifying enzymes, accelerating the metabolism of *Tripterygium wilfordii* toxins and reducing liver damage caused by their toxicity.

#### 3.3.4. Artemisinin (C_15_H_22_O_5_)

Artemisinin is a sesquiterpene lactone compound isolated from *Artemisia annua* L. (Asteraceae), traditionally used to relieve summer-heat (Table 3). Tu Youyou and colleagues discovered that extracts of *Artemisia annua* exerted significant therapeutic effects against rodent and simian malaria [133]. In recent decades, beyond its antimalarial properties, studies have shown that artemisinin and its derivatives possess skin-lightening, anti-glycation, and anti-inflammatory activities [134]; simultaneously, research has been conducted on their anti-tumor, immunomodulatory, and anti-fibrotic effects [135]. In pancreatic cancer, artemisinin downregulates the expression of ferroptosis-related genes such as GPX4, impairing lipid peroxide-scavenging activity; it also promotes the accumulation of intracellular iron ions, which catalyze the production of large amounts of lipid ROS via the Fenton reaction, selectively inducing ferroptosis in damaged PACs and thereby reducing the area of pancreatic necrosis [136].

#### 3.3.5. Andrographolide (C_20_H_30_O_5_)

Andrographolide is a diterpenoid lactone compound isolated from the dried aerial parts (stems and leaves) of *Andrographis paniculata* (Burm. f.) Nees (Acanthaceae) (Table 3). It reduces the expression of the pro-inflammatory factor TNF-α by inhibiting the NF-κB signaling pathway, promotes pancreatic β-cell regeneration, and inhibits adipocyte formation [137]. Andrographolide also directly binds to Ras, blocks GDP-GTP exchange, and inhibits both wild-type and oncogenic KRAS signaling, thereby slowing the progression of pancreatic cancer [138]. Additionally, it reduces Parkinson’s disease protein 7 (DJ-1) expression, leading to the accumulation of ROS, which in turn inhibits the growth of pancreatic cancer cells [139].

**Table 3:** Selected Terpenoids in Acute Pancreatitis: Major Sources and Mechanisms of Action.

Natural Products	Major Sources	Mechanisms	Reference
Triptolide	*Tripterygium wilfordii* Hook. f.	Suppresses STAT3 phosphorylation and lowers IL-6 secretion	[140]
Paeoniflorin	*Paeonia lactiflora* Pall.	Downregulates TMEM16A and reduces the release of TNF-α and IL-1β Downregulates HMGB1/TLR4 signaling pathway Blocks phosphorylation of NF-κB and MAPK	[126,127]
Glycyrrhizic acid	*Glycyrrhiza uralensis* Fisch. ex DC.	Binds HMGB1 and prevents its binding to TLR4 Suppresses MAPK/STAT3/AKT signaling pathway Induces nucleus translocation of Nrf2 to upregulate HO-1, eliminates lipid reactive oxygen species, and inhibits lipid peroxidation Activates the Nrf2-ARE pathway	[130,131,132,141]
Artemisinin	*Artemisia annua* L.	Downregulates ferroptosis-related gene expression	[136]
Andrographolide	*Andrographis paniculata* (Burm. f.) Nees	Blocks NF-κB signaling pathway to decrease TNF-α expression Binds Ras and interrupts GDP-GTP exchange reaction Triggers ROS accumulation to retard fibrosis progression	[137,138,139]

### 3.4. Flavonoids

#### 3.4.1. Baicalein (C_15_H_10_O_5_)

Baicalein is a flavonoid compound extracted from the roots of *Scutellaria baicalensis* Georgi (Lamiaceae). In traditional Chinese medicine, *Scutellaria baicalensis* is used to clear heat and dry dampness, purge fire, and detoxify, primarily for treating lung-heat-induced cough and damp-heat dysentery (Table 4). Recent studies have shown that, in addition to its antioxidant, anti-inflammatory, anti-tumor, hepatoprotective, and neuroprotective activities [142], baicalein exerts multiple protective effects in pancreatitis and associated organ injuries: it inhibits the MAPK/ERK signaling pathway and reduces intracellular ROS levels, thereby alleviating PAC necrosis [143]; in pancreatitis-associated lung injury, it alleviates pulmonary inflammation by upregulating miR-182 and inhibiting TLR4 signaling; and it inhibits the M1 polarization of macrophages via the high-mobility group box 1 protein (HMGB1)/TLR4/NLRP3 pathway, thereby reducing pyroptosis of PAC in hyperlipidemic pancreatitis. Furthermore, baicalein has been found to interfere with ferroptosis [144] and improve intestinal barrier function [145], opening up new prospects for its application in systemic inflammation.

#### 3.4.2. Quercetin (C_15_H_10_O_7_)

Quercetin is a flavonoid compound widely distributed in plants such as acacia seeds, onions, and tea, and has demonstrated significant anti-tumor, anti-inflammatory, and antibacterial activities (Table 4). However, its low water solubility, low stability, and low bioavailability are core challenges to its development [146]. Currently, numerous studies are exploring structural modifications to enhance its bioavailability [147]. In pancreatitis models, quercetin reduces intracellular ROS production at its source by activating the Nrf2/HO-1 antioxidant axis and inhibiting NOX; it also inhibits ERS-induced pro-apoptotic pathways (e.g., protein kinase R-like endoplasmic reticulum kinase (PERK)/C/EBP homologous protein (CHOP)), thereby shifting the mode of PAC death from highly pro-inflammatory necrosis to relatively immunologically silent apoptosis (i.e., enhancing apoptotic cell death) [148], and thereby reducing DAMP leakage and restraining the early inflammatory storm. Additionally, quercetin limits glycolysis by inhibiting PFKFB3, thereby blocking lactate accumulation and excessive activation of the NLRP3/IL-1β inflammasome pathway; this ultimately reduces serum amylase levels and the pancreatic wet-to-dry weight ratio, protecting pancreatic tissue via metabolic regulation [149].

#### 3.4.3. Luteolin (C_15_H_10_O_6_)

Luteolin is a flavonoid widely distributed in plants such as honeysuckle, chrysanthemum, and perilla. In traditional Chinese medicine, these plants are used to disperse wind-heat and clear heat and detoxify (Table 4). Luteolin exhibits a wide range of pharmacological activities, including anti-inflammatory, anti-allergic, anti-tumor, and neuroprotective effects [150,151]. In pancreatitis, luteolin primarily protects pancreatic tissue by regulating the NF-κB mRNA expression, thereby reducing the production of pro-inflammatory factors such as TNF-α and IL-1β [152]; it inhibits the assembly and activation of the NLRP3 inflammasome and caspase-1, reducing the maturation and secretion of IL-1β; it also alleviates pancreatic inflammation indirectly by modulating the gut microbiota and enhancing intestinal barrier function to reduce endotoxin entry into the bloodstream [153]. Regarding the prevention and treatment of pancreatic cancer, Huang et al. demonstrated that luteolin inhibits the formation of tubular complexes and the ectopic expression of cytokeratin-19 during acinar-to-ductal metaplasia, thereby hindering the progression of pancreatic precancerous lesions, suggesting its potential value in this context [154].

#### 3.4.4. Myricetin (C_15_H_10_O_8_)

Myricetin is a flavonoid predominantly distributed in *Ampelopsis grossedentata* (Hand.-Mazz.) *Vitaceae* and the bark and fruit of *Myrica rubra* (Lour.) Sieb. & Zucc. (Myricaceae) (Table 4). In traditional Chinese medicine, *Ampelopsis grossedentata* is used to clear heat and drain dampness, while *Myrica rubra* is used to promote saliva production and quench thirst. Recent studies have highlighted myricetin’s diverse pharmacological benefits, including antioxidant, anti-inflammatory, lipid-lowering, hepatoprotective, nephroprotective effects, and gut microbiota regulatory effects [155]. Although myricetin exhibits favorable safety, and low stability with oral bioavailability limit its clinical application [156]. In AP, myricetin blocks the activation of the NF-κB signaling pathway, downregulating the transcription of pro-inflammatory mediators such as TNF-α and IL-6 to alleviate the inflammatory cascade in PACs; it also restores tight-junction protein expression in PACs, thereby reinforcing the acinar barrier, reducing pancreatic enzyme leakage, and ultimately alleviating the severity of hyperlipidemic pancreatitis [157,158]. Furthermore, myricetin activates the AMPK/SIRT1 signaling axis, promoting AMPK phosphorylation and enhancing SIRT1 deacetylase activity; it also improves tyrosine phosphorylation of insulin receptor substrate-1 (IRS-1) and enhances insulin sensitivity, thereby alleviating insulin resistance and hepatic lipid accumulation [159]. This metabolic regulatory effect provides new insights into protecting against hyperlipidemic pancreatitis injury.

**Table 4:** Flavonoids in Acute Pancreatitis: Major Sources and Mechanisms of Action.

Natural Products	Major Sources	Mechanisms	Reference
Baicalein	*Scutellaria baicalensis* Georgi	Inhibits the MAPK/ERK pathway and reduces intracellular ROS levels Upregulates miR-182, and inhibiting TLR4 suppresses the polarization of macrophages toward the M1 phenotype Interferes with ferroptosis Improves intestinal barrier function	[143,144,145,160]
Quercetin	acacia seeds, onions, and tea	Activates Nrf2/HO-1 and inhibits NADPH oxidase, PERK/CHOP, and other pathways Inhibits PFKFB3	[148,149]
Luteolin	honeysuckle, chrysanthemum, and perilla	Downregulates NF-κB; reduces TNF-α and IL-1β Inhibits the initiation of NLRP3 inflammasome assembly and caspase-1 activation Inhibits the tubular complex formation and aberrant expression of cytokeratin-19	[152,153,154]
Myricetin	*Ampelopsis grossedentata* (Hand.-Mazz.) W. T. Wang, and *Myrica rubra* (Lour.) Sieb. & Zucc.	Inhibits NF-κB, downregulates TNF-α and IL-6, and restores the expression of proteins associated with tight junctions between pancreatic acinar cells Activates the AMPK/SIRT1 signaling axis, promotes AMPK phosphorylation, enhances SIRT1 deacetylation, and improves IRS-1 tyrosine phosphorylation	[157,158,159]

### 3.5. Phenols

#### 3.5.1. Resveratrol (C_14_H_12_O_3_)

Resveratrol is a polyphenolic compound isolated from the roots and rhizomes of *Polygonum cuspidatum* (Polygonaceae), and also widely distributed in grapes, peanuts, and other foods (Table 5). It was first extracted from the roots of *Veratrum grandiflorum* in 1940 [161]. Traditionally, *P. cuspidatum* has been used to promote blood circulation, dissipate blood stasis, clear heat and eliminate dampness. Resveratrol possesses extensive pharmacological activities, including antioxidant, anti-inflammatory, anti-aging, cardiovascular-protective and metabolic-regulatory effects [161,162]. In pancreatitis, resveratrol inhibits NF-κB p65 nuclear translocation and downstream transcriptional activity, reducing the release of pro-inflammatory mediators such as IL-1β and TNF-α and blunting the acinar cell inflammatory cascade [163]; it activates the SIRT1-FOXO1 axis to strengthen SIRT1 deacetylase activity, facilitate FOXO1 nuclear translocation and upregulate autophagy-related genes, thus enhancing autophagic clearance of damaged acinar cells [164]. Furthermore, the SIRT1-FOXO1 axis activation elevates endothelial nitric oxide synthase (eNOS) expression, improves endothelial function and dilates pancreatic microvessels, ameliorating pancreatic microcirculation and tissue perfusion to relieve ischemia-hypoxia-mediated secondary acinar-cell injury [165].

#### 3.5.2. Curcumin (C_21_H_20_O_6_)

Curcumin is a major polyphenolic pigment derived from the rhizomes of Curcuma species (Table 5). Curcuma was first recorded in *Newly Revised Materia Medica*, and has been traditionally applied to resolve blood stasis, promote qi circulation, unblock meridians and relieve pain. Curcumin exhibits multiple pharmacological properties, including anti-inflammatory, antioxidant, anti-fibrotic, antitumor and immunomodulatory effects [166]. Nevertheless, its poor water solubility, prominent first-pass effect and low bioavailability restrict its direct clinical translation [167]. Novel dosage forms, including nanoparticles, micelles, nanoemulsions, liposomes and phospholipid complexes are being developed to optimize its pharmacokinetic profile and therapeutic potency [168]. In pancreatitis, curcumin modulates Keap1 cysteine residues to promote Nrf2 nuclear translocation and ARE binding, upregulating HO-1 and SOD expression to scavenge oxygen free radicals and lipid peroxides, and to counteract oxidative stress triggered acinar cell damage [169]; it suppresses the TGF-β1/Smad2/3 and platelet derived growth factor (PDGF) signalling pathways to inhibit pancreatic stellate cell activation and proliferation, reducing α-SMA expression and excessive extracellular matrix deposition and ultimately alleviating pancreatic oedema and fibrosis [170].

#### 3.5.3. Caffeic Acid (C_9_H_8_O_4_)

Caffeic acid is a phenolic acid abundant in coffee, honeysuckle and *Lithospermum erythrorhizon* (Table 5). It exerts anti-inflammatory, antioxidant, immunomodulatory, antibacterial and microvascular-regulating pharmacological effects [171]. Caffeic acid has a short biological half-life, whereas aspirin can slow its metabolic clearance in rat models [172]. In AP, caffeic acid downregulates C-X-C motif chemokine ligand (CXCL)1 and CXCL2 expression and suppresses neutrophil surface adhesion molecule activation to reduce neutrophil recruitment into pancreatic tissue [173]; it inhibits PAD4 activity and myeloperoxidase release to block NET formation, mitigating NET-derived acinar-cell injury and dampening the local inflammatory cascade [174]. Furthermore, caffeic acid represses the Gasdermin D (GSDMD)-dependent pyroptosis pathway by inhibiting LPS induced caspase-11 activation, blocking GSDMD cleavage and pore forming N-terminal fragment oligomerization to decrease acinar cell content leakage and inflammatory amplification [110]. These anti-inflammatory actions attenuate AP severity, preserve intestinal epithelial-barrier integrity [175] and lower the risk of secondary bacterial translocation-related infection.

**Table 5:** Phenols in Acute Pancreatitis: Major Sources and Mechanisms of Action.

Natural Products	Major Sources	Mechanisms	Reference
Resveratrol	*Polygonum cuspidatum root and rhizome,*	Inhibits NF-κB p65 nuclear translocation, downstream transcription; reduces IL-1β, TNF-α release. Activates SIRT1–FOXO1 axis; upregulates autophagy-related genes; enhances acinar autophagic clearance. Activates eNOS; improves pancreatic microcirculation, tissue oxygen supply.	[161,162,163,164,165]
Curcumin	*Curcuma rootstock*	Modifies Keap1 cysteine residues; activates Nrf2 nuclear translocation, ARE binding; upregulates HO-1, SOD; scavenges ROS, lipid peroxides. Inhibits TGF-β1/Smad2/3, PDGF signaling; inhibits pancreatic stellate cell activation, proliferation; downregulates α-SMA; reduces type I collagen, fibronectin deposition. Poor water solubility, low bioavailability; nanoparticles, liposomes improve pharmacokinetics.	[166,167,168,169]
Caffeic acid	*Plants* such as *coffee, honeysuckle,* and *purple root*	Inhibits CXCL1, CXCL2 expression; inhibits neutrophil adhesion molecule activation, recruitment; inhibits PAD4 activity, MPO release; blocks NET formation. Inhibits GSDMD-mediated pyroptosis; inhibits LPS-induced caspase-11 activation; blocks GSDMD cleavage, membrane pore formation. Protects intestinal epithelial barrier; reduces bacterial translocation risk; short half-life; aspirin delays metabolism.	[171,172,173,174,175]
EGCG, an in vivo hydrolysis product	*Rhus chinensis gall; Camellia sinensis leaf; Quercus infectoria gall*	Scavenges ROS; upregulates HO-1; inhibits oxidative stress-induced acinar apoptosis; protects against L-arginine-induced AP. Activates Nrf2/GPX4 pathway; inhibits acinar ferroptosis; inhibits CYP2E1-mediated ROS production, lipid peroxidation; stabilizes acinar membrane. EGCG potential hepatotoxicity, cardiotoxicity, nephrotoxicity; safety evaluation required.	[176,177,178,179,180,181,182,183]

#### 3.5.4. Gallic Acid (C_7_H_6_O_5_)

Epigallocatechin-3-gallate (EGCG), the characteristic bioactive constituent of tea, can be hydrolyzed to generate gallic acid in vivo [176]. Sharing structural features with polyphenolic hydroxyl, gallic acid and EGCG both exhibit potent antioxidant, antibacterial, anti-inflammatory and antimutagenic activities [177]. Gallic acid is a polyphenolic acid widely distributed in gallnuts, tea, and various plant resources. In AP, gallic acid directly scavenges ROS and upregulates HO 1 expression to enhance antioxidant defense and alleviate oxidative-stress-induced acinar cell apoptosis, conferring pancreatic protection in L arginine-evoked AP models [178]; EGCG activates the Nrf2/GPX4 pathway to enhance antioxidant capacity and suppress acinar cell ferroptosis [179]. Importantly, gallic acid also dose-dependently ameliorates alcohol-provoked pancreatic damage, which has been attributed to inhibition of CYP2E1-mediated ROS generation and lipid peroxidation, stabilization of acinar cell membrane structure, and reduction in pancreatic inflammatory lesions [180]. A study on ethanol-treated rats confirmed that gallic acid (50–200 mg/kg) reduced the release of lysosomal cathepsin B and L into the cytoplasmic fraction and improved ethanol-mediated pancreatic tissue damage. Nevertheless, EGCG is associated with potential hepatotoxicity [181], cardiotoxicity [182] and nephrotoxicity [183], which underscores the necessity of safety evaluation for its clinical translation.

### 3.6. Glycosides Natural Products

#### 3.6.1. Ginsenoside Rb1 (C_54_H_92_O_23_)

Ginsenoside Rb1 is a major bioactive saponin derived from *Panax ginseng*. It exerts prominent pancreatic protective effects against AP injury via dual regulatory mechanisms (Table 6). Ginsenoside Rb1 activates the classic intracellular survival PI3K/Akt signaling pathway [184,185]; activated PI3K/Akt phosphorylates downstream apoptotic proteins including Bad and caspase-9, inhibits mitochondrial cytochrome c release, and blocks the apoptotic cascade, thereby reducing acinar cell apoptosis [186]. In rain frog toxin induced AP models, ginsenoside Rb1 treatment decreases cleaved caspase-3 levels and reduces the number of TdT-mediated dUTP nick-end labeling (TUNEL) positive apoptotic pancreatic cells, verifying its anti-apoptotic activity mediated by apoptotic protein regulation. Furthermore, ginsenoside Rb1 effectively suppresses endoplasmic reticulum stress [186]. Pancreatitis triggers excessive endoplasmic reticulum burden and unfolded protein accumulation in acinar cells, activating PERK, activating transcription factor 6 (ATF6) and immunoglobulin-regulated enhancer 1 (IRE1) stress pathways and inducing CHOP-dependent apoptotic injury. Ginsenoside Rb1 downregulates GRP78 and CHOP expression, relieves endoplasmic reticulum stress, and ultimately mitigates endoplasmic reticulum stress mediated acinar cell apoptosis [187].

#### 3.6.2. Notoginsenoside R1 (C_47_H_80_O_18_)

Notoginsenoside R1 is a core active saponin component extracted from *Panax notoginseng* (Table 6). It ameliorates systemic inflammatory responses in severe acute pancreatitis primarily through gut microbiota modulation [188]. Notoginsenoside R1 remodels gut microbiota structure and composition, enriches short chain fatty acid producing bacteria, and promotes the synthesis of butyrate and propionate [188]. The accumulated short-chain fatty acids nourish intestinal epithelial cells, maintain intestinal mucosal barrier integrity, reduce intestinal permeability, and inhibit endotoxin translocation. Additionally, notoginsenoside R1 selectively enriches mucin-degrading microbiota, suppresses proinflammatory cytokines IL-6 and TNF-α, and upregulates the anti-inflammatory cytokine IL-10 [189]. This gut-targeted regulatory mechanism blocks the dissemination of gut-derived systemic inflammation, thereby alleviating severe pancreatitis-associated systemic inflammatory responses. It complements conventional anti-inflammatory pharmacological strategies and provides a novel therapeutic direction for severe pancreatitis treatment.

#### 3.6.3. Astragaloside IV (C_41_H_68_O_14_)

Astragaloside IV is the predominant active saponin constituent of *Astragalus membranaceus*, and it exerts pancreatic protective effects by inhibiting inflammatory signaling and delaying pancreatic fibrosis progression (Table 6). Astragaloside IV suppresses inflammatory responses via the TLR4/MyD88/NF-κB pathway, significantly reducing the expression of TLR4, MyD88 and NF-κB [190]. In AP models, it markedly decreases NF-κB nuclear staining intensity in pancreatic tissue by restraining NF-κB nuclear translocation [191]. Furthermore, astragaloside IV regulates the TRAF6/NF-κB pathway to promote the secretion of miR-146a-5p-enriched exosomes by mesenchymal stem cells [192]. The secreted miR-146a-5p targets TLR4, IRAK1 and TRAF6 to further inhibit excessive NF-κB activation. These synergistic anti-inflammatory effects alleviate pancreatic inflammatory damage and hinder the progression of chronic pancreatitis and pancreatic fibrosis.

#### 3.6.4. Rutin (C_27_H_30_O_16_)

Rutin is a ubiquitous flavonoid glycoside (quercetin-3-O-rutinoside) distributed in *Rutaceae* and *Ricinaceae* plants, with potent antioxidant activity and protective effects on pancreatic exocrine function in alcoholic pancreatitis (Table 6). Ethanol metabolism generates massive ROS and depletes endogenous antioxidant systems, a process that constitutes the core pathogenesis of alcoholic pancreatitis. Rutin enhances the activities of superoxide dismutase and catalase to improve endogenous antioxidant capacity [193]. In caerulein toxin- and L-arginine induced pancreatitis models, rutin reduces pancreatic malondialdehyde levels, elevates superoxide dismutase activity and increases reduced glutathione content, thereby eliminating excessive oxidative stress [194]. These antioxidant effects collectively protect pancreatic exocrine function against pancreatitis injury [195,196,197]. As a safe and accessible dietary flavonoid, rutin possesses great potential for the prevention and adjuvant treatment of alcoholic pancreatitis.

**Table 6:** Glycosides in Acute Pancreatitis: Major Sources and Mechanisms of Action.

Natural Products	Major Sources	Mechanisms	Reference
Ginsenoside	*ginseng*	Activates PI3K/Akt survival pathway; phosphorylates Bad, caspase-9; inhibits mitochondrial cytochrome c release; blocks apoptotic cascade. Inhibits cleaved caspase-3 expression; reduces TUNEL-positive apoptotic acinar cells. Inhibits GRP78, CHOP expression; inhibits PERK, ATF6, IRE1 endoplasmic reticulum stress pathways; alleviates acinar apoptotic damage.	[185,186,187]
*Panax notoginseng* saponins	*pseudo-ginseng*	Regulates gut microbiota composition; activates short-chain fatty acid-producing bacteria; promotes butyric acid and propionic acid generation; maintains intestinal mucosal barrier integrity; reduces intestinal permeability, endotoxin translocation. Enriches mucin-degrading microbiota; inhibits IL-6, TNF-α expression; activates IL-10 expression; blocks gut-derived systemic inflammatory signals; alleviates severe acute pancreatitis systemic inflammation.	[188,189]
Astragaloside IV	*Astragalus mongholicus*	Inhibits TLR4/MyD88/NF-κB inflammatory pathway; inhibits NF-κB nuclear translocation; reduces pancreatic inflammatory injury. Regulates TRAF6/NF-κB pathway; activates mesenchymal stem cell exosome secretion with miR-146a-5p; inhibits TLR4, IRAK1, TRAF6 expression; suppresses excessive NF-κB activation. Alleviates pancreatic inflammation; delays fibrotic progression of chronic pancreatitis.	[190,191,192]
Rutin	*Plants from the Rutaceae* and *Ricinaceae families, among others*	Activates superoxide dismutase, catalase activity; increases reduced glutathione level; inhibits malondialdehyde accumulation; scavenges ROS; inhibits ethanol-induced oxidative stress injury. Protects pancreatic exocrine function; presents high safety and broad application prospects for alcoholic pancreatitis prevention and adjuvant treatment.	[194,195,196,197]

### 3.7. Other Miscellaneous Compounds

#### 3.7.1. Allicin (C_6_H_10_OS_2_)

A sulfur-containing compound extracted from garlic, allicin is one of the primary active components. In traditional medicine, garlic has been used to detoxify, reduce swelling, kill parasites, and stop diarrhea (Table 7). Modern research has confirmed its broad-spectrum antibacterial and anti-inflammatory activities. In a caerulein-induced acute pancreatitis mouse model, the garlic-derived organic sulfur compound diallyl disulfide (DADS) inhibited IκB degradation and NF-κB nuclear translocation, reduced serum amylase and myeloperoxidase activities, and attenuated pathological damage in pancreatic and lung tissues [198].

Another study demonstrated that DADS exerts its anti-inflammatory effects by activating the PPAR-γ pathway, inhibiting STAT3 and NF-κB activation, and downregulating the expression of inflammatory cytokines such as TNF-α [199]. A 2025 study further confirmed that allicin itself protects pancreatic tissue from injury by inhibiting NF-κB transcription and ATF4 phosphorylation, thereby alleviating endoplasmic reticulum (ER) stress and oxidative stress [200].

#### 3.7.2. Tea Polyphenols

A general term for polyphenolic compounds in tea, primarily consisting of catechins, tea polyphenols are the major bioactive constituents of this beverage (Table 7). Traditionally, tea has been used to clear the mind, relieve irritability, promote digestion, reduce phlegm, promote diuresis, and detoxify. In a cerulein-induced acute pancreatitis mouse model, green tea polyphenols reduced serum amylase and lipase levels and attenuated pancreatic histopathological injury. The underlying mechanism involves inhibition of NF-κB activation and MAPK (ERK, JNK, p38) phosphorylation, as well as downregulation of inflammatory cytokines including TNF-α, IL-1β, and IL-6 [201]. A systematic review and meta-analysis showed that EGCG protects against acute pancreatitis in animal models by regulating NF-κB, JAK/STAT, and Nrf2 signaling cascades [202].

#### 3.7.3. Lycopene (C_40_H_56_)

A natural carotenoid found primarily in tomatoes, watermelon, and other fruits and vegetables, lycopene is one of the most potent antioxidants known for scavenging singlet oxygen (Table 7). A review noted that lycopene exerts its anti-inflammatory effects through its antioxidant activity, inhibition of inflammatory cytokine expression, and reduction in cell death via activation of AMPK-dependent autophagy, thereby ameliorating pancreatitis [203]. In cerulein-stimulated pancreatic acinar cells, lycopene inhibited STAT3 phosphorylation and reduced IL-6 expression and secretion [203].

Mechanistic studies further demonstrated that cerulein activates NADPH oxidase to produce ROS, which in turn activate NF-κB and induce inflammatory cytokine expression. Lycopene blocks this inflammatory signaling pathway by scavenging ROS and inhibiting NADPH oxidase activity, thereby suppressing the production of cytokines such as IL-6 [204].

#### 3.7.4. Astaxanthin (C_40_H_52_O_4_)

**Figure 5:**
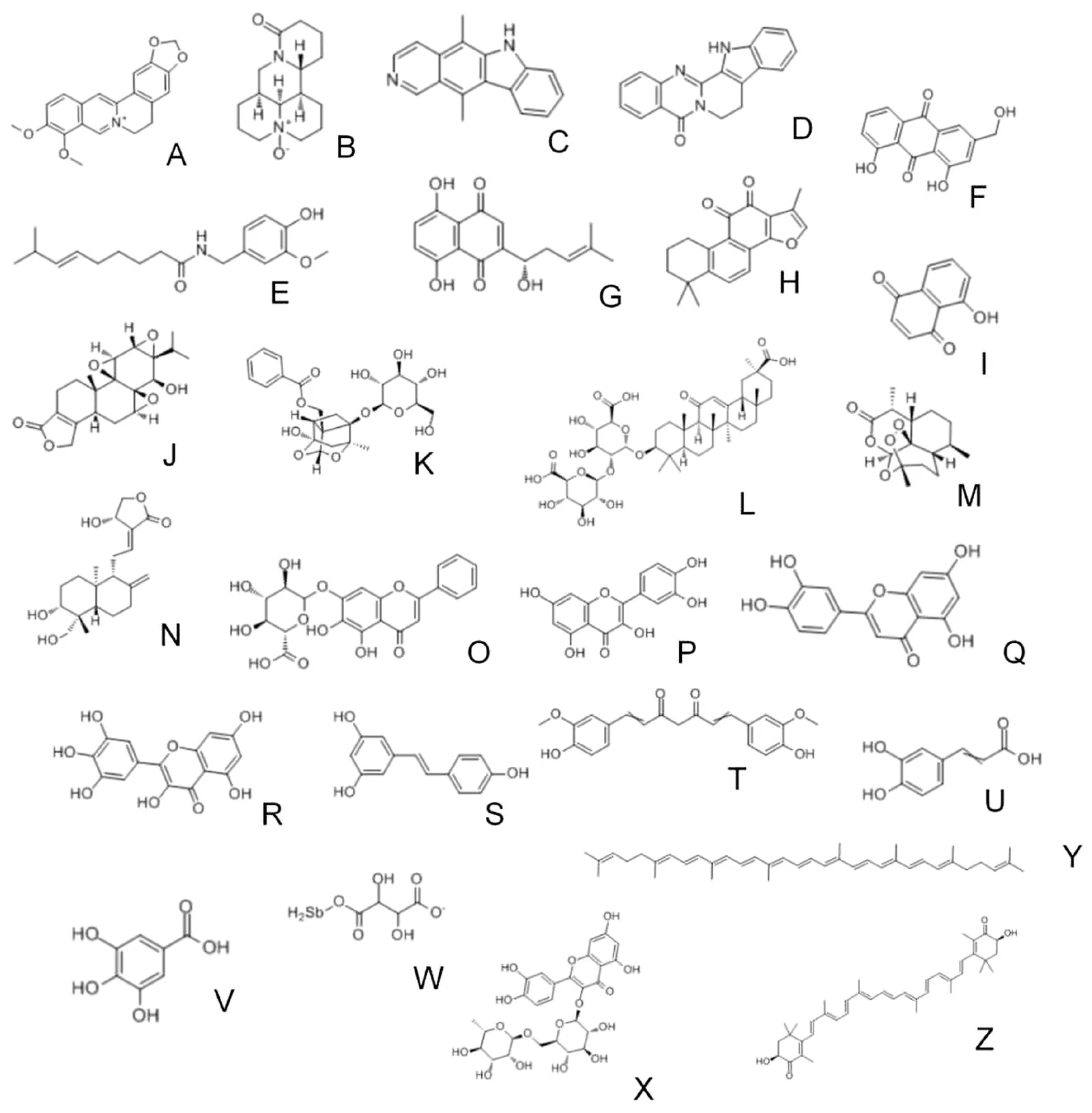
Chemical structures of different classes of compounds. (**A**): Berberine; (**B**): oxymatrine; (**C**): Ellipticine; (**D**): Evodiamine; (**E**): Capsaicin; (**F**): Emodin; (**G**): Shikonin; (**H**): Tanshinone IIA; (**I**): Juglone; (**J**): Triptolide; (**K**): Paeoniflorin; (**L**): Glycyrrhizic acid; (**M**): Artemisinin; (**N**): Andrographolide; (**O**): Baicalein; (**P**): Quercetin; (**Q**): Luteolin; (**R**): Myricetin; (**S**): Resveratrol; (**T**): Curcumin; (**U**): Caffeic acid; (**V**): Gallic acid; (**W**): Astragaloside IV; (**X**): Rutin; (**Y**): Lycopene; (**Z**): Astaxanthin. (http://www.ichemistry.cn/).

A natural ketocarotenoid found primarily in shrimp, crab, salmon, and other aquatic products, as well as in the microalga *Haematococcus pluvialis*, it possesses potent antioxidant activity (Table 7). A study showed that astaxanthin improves defective autophagy in acute pancreatitis via Nrf2 activation. This compound also inhibits formation of the Beclin1-Slc7a11 complex, thereby suppressing ferroptosis in acinar cells and alleviating pancreatic pathological injury [205]. Another study indicated that astaxanthin can activate Nrf2 and inhibit NF-κB nuclear translocation, reducing pro-inflammatory cytokine expression and enhancing antioxidant enzyme activity [206] (Figure 5).

**Table 7:** Miscellaneous natural compounds in acute pancreatitis: major sources and mechanisms of action.

Natural Products	Major Sources	Mechanisms	Reference
Allicin/DADS (Garlic-derived organosulfur compound)	*Garlic (Allium sativum)*	Inhibits IkB degradation and NF-B nuclear translocation Activates PPAR-y pathway; suppresses STAT3 and NF-KB activation; Reduces TNF-a and other inflammatory cytokines Inhibits NF-κB transcription and ATF4 phosphorylation; alleviates ER stress and oxidative stress	[198,199,200]
Tea polyphenols (EGCG)	*Tea leaves (Camellia sinensis)*	Inhibits NF-kB activation and MAPK (ERK, JNK, p38) phosphorylation; Downregulates TNF-a, IL-1ß, IL-6 expression Regulates NF-kB, JAK/STAT, and Nrf2 signaling pathways	[201,202]
Lycopene	*Tomatoes, watermelon, and other red fruits/vegetables*	Activates AMPK-dependent autophagy; reduces acinar cell death Inhibits STAT3 phosphorylation; reduces IL-6 expression and secretion Scavenges ROS and inhibits NADPH oxidase activity; blocks NF-κB activation	[203,204]
Astaxanthin	*Shrimp, crab, salmon, and microalga Haematococcus pluvialis*	Activates Nrf2; improves defective autophagy Inhibits Beclin1-Slc7a11 complex formation; suppresses acinar cell ferroptosis Inhibits NF-κB nuclear translocation; reduces pro-inflammatory cytokines and enhances antioxidant enzyme activity	[205,206]

## 4. Preclinical Advances in Natural Product-Based Prevention and Treatment of Pancreatitis

Extensive in vitro and in vivo evidence has established that a wide spectrum of naturally occurring bioactive compounds-including flavonoids, terpenoids, anthraquinones, and alkaloids-exert pronounced protective effects against pancreatic injury in experimental models of AP, SAP, and CP. These effects operate through intricately interconnected regulatory networks that modulate oxidative stress, inflammatory signaling, and cell death pathways [60].

At the combination therapy level, co-administration of resveratrol with myrrh sterols or β-carotene has been shown to yield synergistic anti-inflammatory and antioxidant outcomes. Innovative delivery strategies, such as emodin-loaded mesenchymal stem-cell-derived extracellular vesicles and nanoparticle-based co-delivery systems incorporating proanthocyanidins with pentoxifylline, have further enhanced target specificity and therapeutic potency [207,208]. Nevertheless, the translational relevance of these findings remains constrained by their near-exclusive reliance on immortalized cell lines and rodent models. Moreover, classic drug development hurdles—poor aqueous solubility and suboptimal oral bioavailability—persist as formidable barriers to clinical adoption.

### 4.1. Engineering Applications of Plant-Derived Compounds in Pancreatitis Management

Nanocarrier platforms offer a refined approach to the targeted delivery of phytochemicals. Lipid-based, polymeric, and macromolecular nanoparticles can efficiently direct active natural products to pancreatic lesions via either passive (enhanced permeability and retention, EPR-mediated) or active targeting mechanisms [209,210], while simultaneously prolonging the circulatory half-life of the payload [211] and reducing serum levels of pancreatic injury biomarkers [212]. Cell membrane-coated nanoparticles, which leverage intrinsic homing affinities, represent a particularly promising biomimetic strategy for lesion-specific accumulation [213]. Lipoprotein carriers, polymeric micelles, and dendrimers have been demonstrated to enhance the intestinal solubilization of hydrophobic phytochemicals, thereby improving oral bioavailability [214]. Inorganic nanoparticles, though capable of mimicking endogenous antioxidant enzyme activities [215], raise safety concerns due to evidence that intravenous administration may potentiate metastatic progression in malignant settings—a limitation that narrows their clinical window [216]. Collectively, these carrier systems provide versatile platforms for the pancreatic-directed delivery of natural products, enabling sustained modulation of local inflammatory cascades.

Hydrogels and microneedle patch systems represent localized intervention modalities that diversify the clinical applicability of phytochemicals. Hydrogels preserve native extracellular matrix architecture and exhibit favorable biocompatibility profiles [217]. When loaded with active plant-derived compounds [218], they offer a promising strategy for regional modulation of fibrotic remodeling and inflammatory progression in CP, although empirical data in this context remain scarce. In parallel, microneedle patches enable painless, minimally invasive transdermal administration that circumvents first-pass hepatic metabolism [219,220,221]. This route facilitates sustained intra-pancreatic retention of bioactives, permitting dose-sparing regimens that markedly reduce systemic drug exposure and off-target toxicity [222]—features that render this platform attractive for both pancreatitis and pancreatic cancer applications.

Probiotics and synthetic biology approaches are advancing the phytochemical field through two complementary avenues: synergistic bioactivity enhancement and scalable biomanufacturing. Genetically engineered probiotics can potentiate the effects of plant-derived compounds; for instance, engineered lactic acid bacteria co-administered with resveratrol have been shown to produce therapeutic metabolites in situ within the gut, while concurrently modulating the intestinal microbiome and reinforcing mucosal barrier integrity [223,224], and these effects collectively ameliorating pancreatitis severity. Separately, CRISPR-based engineering of yeast and *E. coli* has enabled the development of high-yield microbial cell factories capable of producing plant-derived natural products at industrial scale [225,226]. This biotechnological strategy substantially lowers production costs and ensures a consistent, quality-controlled supply, thereby addressing a critical bottleneck in the translational pathway of phytotherapeutic agents.

### 4.2. Future Directions for Natural Product-Based Approaches to Pancreatitis Treatment

Future research on natural product-based strategies for managing pancreatitis will primarily focus on three key areas: AI-assisted drug discovery, gut–pancreas axis-targeted regulation, and personalized precision medicine. The first area involves leveraging artificial intelligence to accelerate new drug discovery—utilizing machine learning algorithms to rapidly screen vast libraries of natural compounds for those capable of targeting key pathways involved in pancreatitis, followed by the use of molecular simulation techniques to identify lead compounds. To date, AI has enabled the discovery of novel active compounds such as glycyrrhizin C, while also facilitating the optimization of dosages and administration protocols for known pharmaceuticals like resveratrol; furthermore, the AI-driven approach to repurposing existing drugs can uncover new applications for anti-pancreatitis therapies derived from safe natural substances, thereby significantly enhancing overall R&D efficiency [227,228]. Second, there is a focus on delving deeper into the mechanistic links between the gut and the pancreas: research has revealed that beneficial gut microbiota and their metabolites can indirectly mitigate pancreatic injury by repairing the intestinal barrier and modulating inflammatory signaling; future work will aim to elucidate how natural products protect the pancreas by regulating the gut microbiota, and will involve the development of specialized natural product formulations designed for intestinal release to precisely target the entero-pancreatic axis [229,230]. Third, efforts are advancing toward personalized precision medicine, where dosing regimens are customized based on individual patient genetic variations and gut microbiota profiles; simultaneously, micro-pancreatic organoids cultured from the patient’s own cells-are being used to test drug efficacy. Currently, organoid models for chronic pancreatitis have been established, and these models will be extended to acute pancreatitis in the future to enable high-throughput screening of natural products and evaluation of their safety, thereby forming a comprehensive pipeline encompassing AI-based initial screening, mechanism validation, and organoid-based drug testing, thereby accelerating the translation of natural products from laboratory research to clinical application [231].

### 4.3. Natural Products in Chronic Pancreatitis

The therapeutic logic for natural products in CP differs fundamentally from that in AP. Whereas AP is dominated by acinar cell injury, premature enzyme activation, and acute inflammatory cascades, CP is driven primarily by the persistent activation of PSCs, leading to ECM deposition and progressive pancreatic fibrosis. Accordingly, the most relevant natural products for CP are those that directly suppress PSC activation and fibrogenesis, rather than those solely targeting acinar cell death or acute oxidative burst.

Among the best-characterized compounds, baicalin has been shown to ameliorate pancreatic fibrosis in cerulein-induced CP mice by inhibiting PSC activation through the TGF-β1/TGF-βR1/TAK1/NF-κB axis, reducing α-SMA expression, collagen deposition, and macrophage infiltration. Rhein, an anthraquinone derivative, attenuates PSC activation and fibrosis in experimental CP by suppressing SHH/GLI1 signaling and downregulating α-SMA, TGF-β, fibronectin, and type I collagen. Resveratrol orally administered to CP mice reduces PSC activation, ECM deposition, and acinar atrophy, with mechanisms involving nuclear Mist1 accumulation and negative modulation of Akt and p38 MAPK pathways. Curcumin has similarly been reported to inhibit PSC proliferation via HO-1 induction and ERK1/2 suppression in cultured PSCs, and a study further demonstrated that curcumin ameliorates cerulein-induced CP through Nrf2/HO-1 signaling. These findings collectively position PSC-directed anti-fibrotic activity as the principal mechanistic theme for natural products in CP [169,232,233,234].

However, the evidence base for CP remains substantially narrower than for AP. Most studies are confined to rodent models, with limited dose-response characterization, scarce pharmacokinetic data, and no clinical trials of natural products specifically in CP. The heterogeneity of CP models and the lack of standardized fibrosis endpoints further complicate cross-study comparison. Thus, while the PSC-targeting effects of baicalin, rhein, resveratrol, and curcumin provide a rational foundation, their translational potential in CP should be framed as emerging and hypothesis-generating (Table 8).

### 4.4. Challenges and Strategies for the Prevention and Treatment of Pancreatitis Using Natural Products

Natural products exhibit considerable potential for the prevention and treatment of pancreatitis. However, many identified components currently suffer from low bioavailability and poor stability. For instance, in studies on the use of polyphenols for treating pancreatitis, the concentrations used in vitro are often far higher than those achievable in humans. Additionally, these compounds exhibit low absorption in the human gastrointestinal tract and are rapidly metabolized and excreted, preventing them from persisting in the pancreas for a sufficient duration [246]. Novel strategies—including nanodrug delivery systems [247], cocrystal technology [248], and prodrug design [249]—hold promise for overcoming this challenge.

In addition, although the pharmacological mechanisms of natural products against pancreatitis are gradually being clarified, their multi-target characteristics and complex crosstalk in signaling networks hinder the accurate identification of core active components and functional pathways. Network pharmacology resolves this problem by constructing compound-target-disease interaction networks to systematically analyze the synergistic mechanisms of multi-component natural products. For instance, network pharmacology-based analysis has clarified that Glycyrrhiza glabra alleviates AP via the regulation of MAPK and STAT3 signaling pathways [61]. Affinity-based target identification is another effective strategy; labeled natural compounds are used as molecular probes to capture binding proteins and screen potential therapeutic targets [19]. Gene-knockout animal models further validate the functional roles of key targets in pancreatitis pathogenesis. For example, C57BL/6N-background heterozygous Spink1 knockout mice have been generated to confirm the causal relationship between SPINK1 mutation and the initiation and progression of CP [250]. Variability in dosing and administration routes: Dosing regimens in animal studies vary widely and are rarely justified by pharmacokinetic data. Rodent models, particularly mice and rats, remain the dominant platform for pancreatitis research, yet the anatomy, physiology, and molecular cell biology of the rodent pancreas differ substantially from those of humans, especially with respect to ductal function. The apparent tension between multi-target activity and target specificity is therefore best resolved not by abandoning multi-target agents, but by applying network pharmacology, proteomics, and genetic validation to define the essential target subset for each compound in each disease context. Lack of standardization in natural product extracts. Most preclinical studies administer crude or partially purified extracts whose chemical composition is incompletely defined. Standardization typically relies on one or a few marker compounds, which provides only partial quality assurance and does not capture the full phytochemical profile or the potential contribution of unmeasured constituents.

Finally, insufficient clinical evidence remains the major translational gap in this field. The anti-pancreatitis efficacy of most natural products is only validated by in vitro and animal studies, with a lack of high-quality clinical trials [60]. For example, curcumin exhibits prominent anti-inflammatory effects in multiple pancreatitis animal models, while large-scale randomized controlled trials (RCTs) are still lacking that verify its clinical efficacy in patients [251]. Rigorous Phase II/III clinical trials designed according to randomization, control, blinding, and reproducibility principles are urgently needed to objectively evaluate the clinical efficacy, recurrence rate, and quality-of-life improvement of natural product-based therapies. Notably, clinical investigations of natural products have been conducted in multiple diseases: hydroxysafflor yellow A (HSYA) has been tested for atherosclerosis [252], and various natural products have been evaluated for osteoarthritis treatment. In pancreatitis research, Chaiqin Chengqi decoction (CQCQD) and *Rheum palmatum* L. have also undergone preliminary clinical evaluation, providing valuable references for subsequent translational studies [253,254].

## 5. Conclusions

No definitive targeted therapy is currently available for pancreatitis, and existing clinical management mainly relies on supportive treatment. With increasing recognition of the pharmacological value of traditional Chinese herbal medicines, natural products have been widely demonstrated to exert prominent anti-inflammatory and pancreatic protective effects, showing great potential for pancreatitis prevention and treatment. Current research has evolved from single compound validation to integrated investigations combining mechanistic exploration and technological innovation.

Future progress in this field will rely on five key research directions: (i) systematic target screening based on network pharmacology; (ii) optimization of novel structural modification and advanced delivery systems for natural products; (iii) mechanistic exploration of the gut–pancreas axis; (iv) construction of personalized precision medicine frameworks; and (v) implementation of rigorous prospective clinical trials. The integration of these strategies will accelerate the development of next-generation pancreatitis therapeutic regimens. Collectively, the combination of classical pharmacological theories and emerging biotechnologies will substantially promote the translational pipeline from basic discovery to clinical application, providing safer and more effective therapeutic options for pancreatitis patients.

## Figures and Tables

**Table 8:** Comparative pharmacokinetic, toxicological, and developmental status of natural products in pancreatitis.

Compound	Toxicity/Safety	Experimental Dose & Route	Bioavailability/PK	Development Stage	Refs.
Curcumin	High-dose animal studies show reversible gastric erosion and mild hepatic fatty changes; human trials with nano-formulation report no serious adverse effects	Animal: 100 mg/kg p.o. or 7.5 mg/kg i.p.; Human: 40 mg/day nano-curcumin (AP RCT); 400 mg nano vs. 2000 mg free (PK trial)	Free curcumin: extremely low systemic exposure; nano-formulations significantly enhance AUC	Clinical: RCT in mild-moderate AP (*n* = 42, reduced LOS and analgesic need); healthy volunteer PK	[235]
Resveratrol	Generally well tolerated in human trials; no serious adverse events at doses up to 5 g	Human: 25–5000 mg p.o. (PK studies); pancreatitis-specific dosing not established	Oral absorption ≥70%, but extensive first-pass sulfate/glucuronide conjugation; unchanged resveratrol <5 ng/mL in plasma	Preclinical only in pancreatitis; registered trial for post-ERCP prevention (NCT02947932) not yet reported	[236]
Quercetin	Limited pancreatitis-specific safety data	Animal: 50 mg/kg p.o. (rat PK); human: 500 mg (PK reference only)	Rat: Cmax 0.26 μmol/L at 50 μmol/kg; human: Cmax 0.8 μmol/L at 500 mg (aglycone)	Preclinical only (AP/CP animal models)	[237]
Baicalin/Baicalein	Not reported in pancreatitis models	Animal: in situ perfusion studies; no pancreatitis-specific dose established	Baicalein well absorbed in stomach/small intestine; baicalin poorly absorbed in intestine; baicalein preferred for oral dosing	Preclinical only (CP animal models)	[238]
Rhein	Hepatotoxicity documented: mitochondrial dysfunction, GSH depletion, lipid peroxidation in rat hepatocytes (LD50 20 μM)	Animal: 50 mg/kg/day p.o. (CP model); human PK: rhubarb extract 50 mg/kg p.o.	Free rhein: Cmax 1.96 μg/mL, AUC 7.32 μg·h/mL; SNEDDS formulation: Cmax 8.0 μg/mL, AUC 37.79 μg·h/mL (4–5× improvement)	Preclinical + preliminary clinical (*Rheum palmatum*); no RCT	[239]
Emodin	Dose- and context-dependent hepatotoxicity and nephrotoxicity; sex-dependent PK differences	Animal: 20–40 mg/kg p.o.; 5 mg/kg i.v. (rat PK)	Absolute bioavailability ~3.2% in rats; extensive first-pass glucuronidation; parent emodin undetectable after oral dosing	Preclinical only	[240,241]
Shikonin	Potential nephrotoxicity and skin allergy; safety not fully defined	Pancreatitis-specific dosing not established	Unfavorable oral bioavailability; plasma protein binding 64.6%; induces CYP450	Preclinical only	[242,243,244]
Paeoniflorin	Not reported in pancreatitis models	Pancreatitis-specific dosing not established	Rat absolute oral bioavailability 3.2–3.8%; Glycyrrhiza protein nanoparticles enhance exposure	Preclinical only	[245]

## Data Availability

No new data were created or analyzed in this study. Data sharing is not applicable to this article.

## Abbreviations

The following abbreviations are used in this manuscript:

AP	acute pancreatitis
CP	chronic pancreatitis
PC	pancreatic cancer
NSAIDs	nonsteroidal anti-inflammatory drugs
ERCP	endoscopic retrograde cholangiopancreatography
CYP2E1	Cytochrome P450 family 2 subfamily E member 1
NADPH	Nicotinamide Adenine Dinucleotide Phosphate Hydrogen
NOX	NADPH oxidase
PACs	pancreatic acinar cells
ROS	reactive oxygen species
mtDNA	mitochondrial DNA
IL-6	Interleukin-6
IL-1β	Interleukin-1β
TNF-α	Tumor Necrosis Factor-alpha
NF-κB	nuclear factor kappa-B
MAPK	mitogen-activated protein kinase
JNK	c-Jun N-terminal kinase
ERK	extracellular regulated protein kinases
SOD	superoxide dismutase
GSH	glutathione
GPx	glutathione peroxidase
APE1	Apurinic/apyrimidinic Endonuclease 1
Ref-1	redox-factor-1
Trx1	thioredoxin 1
MDA	malondialdehyde
Nrf2	nuclear factor erythroid 2-related factor 2
NLRP3	NOD-like receptor thermal protein domain associated protein 3
DAMPs	damage-associated molecular patterns
PAMPs	pathogen-associated molecular patterns
TLR4	Toll-like receptor 4
LPS	Lipopolysaccharides
MyD88	myeloid differentiation primary response protein 88
STAT3	signal transducer and activator of transcription 3
TRIF	TIR domain-containing adaptor inducing interferon-β
MCP-1/CCL2	monocyte chemoattractant protein-1
SIRS	systemic inflammatory response syndrome
MMPs	matrix metalloproteinases
ECM	extracellular matrix
NETs	neutrophil extracellular traps
AHR	aryl hydrocarbon receptor
LAMP-2	lysosome-associated membrane protein 2
ER	endoplasmic reticulum
DISC	death-inducing signaling complex
mPTP	mitochondrial permeability transition pore
CHOP	C/EBP Homologous Protein
ATP	Adenosine Triphosphate
PI3K	Phosphatidylinositol 3-kinase
AKT	Protein Kinase B
Ca^2+^	calcium ions
IP3Rs	inositol 1,4,5-trisphosphate receptors
RyRs	ryanodine receptors
Orai1	calcium release-activated calcium channel protein 1
NFAT	nuclear factor of activated T cells
UPR	unfolded protein response
PSCs	pancreatic stellate cells
PRSS1/2	serine protease 1/2
SPINK1	serine peptidase inhibitor Kazal type 1
CTRC	chymotrypsin C
CPA1	carboxypeptidase A1
CEL	carboxyl ester lipase
CFTR	cystic fibrosis transmembrane conductance regulator
CLDN2	claudin-2
CASR	calcium-sensing receptor
KRAS	KRAS proto-oncogene
UCP2	uncoupling protein 2
SIRT3	Sirtuin 3
PGC1α	Peroxisome Proliferator-Activated Receptor Gamma Coactivator 1-alpha
CGRP	calcitonin gene-related peptide
TRPV1	transient receptor potential vanilloid 1
PPARγ	peroxisome proliferator-activated receptor gamma
SAP-ALI	severe acute pancreatitis-associated acute lung injury
VDAC1	voltage-dependent anion channel 1
TanIIA	Tanshinone IIA
SOD2	Superoxide dismutase
PDAC	pancreatic ductal adenocarcinoma
DJ-1	Parkinson’s disease protein 7
HMGB1	high mobility group box 1 protein
HO-1	Heme Oxygenase-1
PERK	Protein Kinase R-like Endoplasmic Reticulum Kinase
IRS-1	insulin receptor substrate-1
eNOS	endothelial nitric oxide synthase
PDGF	platelet-derived growth factor
CXCL	C-X-C motif chemokine ligand
GSDMD	Gasdermin D
EGCG	Epigallocatechin-3-gallate
TUNEL	TdT-mediated dUTP Nick-End Labeling
ATF6	Activating Transcription Factor 6
IRE1	Immunoglobulin-Regulated Enhancer 1
DADS	diallyl disulfide
NADPH	Nicotinamide Adenine Dinucleotide Phosphate
CQCQD	Chaiqin Chengqi Decoction
